# Clonality and Evolutionary History of Rhabdomyosarcoma

**DOI:** 10.1371/journal.pgen.1005075

**Published:** 2015-03-13

**Authors:** Li Chen, Jack F. Shern, Jun S. Wei, Marielle E. Yohe, Young K. Song, Laura Hurd, Hongling Liao, Daniel Catchpoole, Stephen X. Skapek, Frederic G. Barr, Douglas S. Hawkins, Javed Khan

**Affiliations:** 1 Genetics Branch, Oncogenomics Section, Center for Cancer Research, National Cancer Institute, National Institutes of Health, Bethesda, Maryland, United States of America; 2 Pediatric Oncology Branch, Center for Cancer Research, National Institutes of Health, Bethesda, Maryland, United States of America; 3 Biospecimens Research and Tumour Bank, The Kids Research Institute, The Children's Hospital at Westmead, Westmead, New South Wales, Australia; 4 Department of Pediatrics, Division of Pediatric Hematology/Oncology, UT Southwestern Medical Center, Dallas, Texas, United States of America; 5 Laboratory of Pathology, Center for Cancer Research, National Cancer Institute, National Institutes of Health, Bethesda, Maryland, United States of America; 6 Department of Pediatrics, Seattle Children’s Hospital, Fred Hutchinson Cancer Research Center, Seattle, Washington, United States of America; St. Jude Children's Research Hospital, United States of America

## Abstract

To infer the subclonality of rhabdomyosarcoma (RMS) and predict the temporal order of genetic events for the tumorigenic process, and to identify novel drivers, we applied a systematic method that takes into account germline and somatic alterations in 44 tumor-normal RMS pairs using deep whole-genome sequencing. Intriguingly, we find that loss of heterozygosity of 11p15.5 and mutations in RAS pathway genes occur early in the evolutionary history of the PAX-fusion-negative-RMS (PFN-RMS) subtype. We discover several early mutations in non-RAS mutated samples and predict them to be drivers in PFN-RMS including recurrent mutation of *PKN1*. In contrast, we find that PAX-fusion-positive (PFP) subtype tumors have undergone whole-genome duplication in the late stage of cancer evolutionary history and have acquired fewer mutations and subclones than PFN-RMS. Moreover we predict that the *PAX3-FOXO1* fusion event occurs earlier than the whole genome duplication. Our findings provide information critical to the understanding of tumorigenesis of RMS.

## Author Summary

To decipher the dynamic mutational process and identify the causative genomic events in rhabdomyosarcoma (RMS), we developed a systematic method that incorporates multiple types of genomic information to estimate normal cell contamination, tumor clonality, and a timeline of somatic events that occurred prior to the tumor presentation. Our results demonstrate two distinct evolutionary paths resulting in PAX-fusion-negative-rhabdomyosarcoma (PFN-RMS) and PAX-fusion-positive-rhabdomyosarcoma (PFP-RMS): (1) In PFN-RMS, genomic loss of heterozygosity on chromosome 11p15.5 and non-synonymous mutations in RAS pathway and cell cycle genes (*FGFR4*, *KRAS*, *NRAS*, *HRAS* and *CCDN1*), as well as several genes not previously known to be drivers of RMS, including *PKN1*, *CUL2*, and *TTK*, occurs early in the evolutionary history of tumor; (2) In contrast, the PAX gene fusion event in PFP-RMS tumors is an early detectable event which consistently occurs prior to a whole genome duplication event. These findings provide new insights into the biology and molecular events that initiate RMS tumorigenesis and may help identify actionable drivers for targeted therapies.

## Introduction

Cancer development is driven by dynamic mutational processes and selective pressures which allow a tumor to adapt over time from the initiating oncogenic lesion towards clinical presentation [1–4]. High coverage, next-generation sequencing technologies have provided an unprecedented view of the mutational landscape of whole cancer genomes and demonstrated that cancer genomes have typically acquired thousands of somatic alterations by the time they are clinically detected [5–8]. Although a majority of these alterations do not have clear biological consequences, some alterations are recurrently found; implicating them as critical events in that tumor’s evolution. Subsequently, external factors such as the tumor microenvironment and therapy can confer selective advantages that allow successful clones to eventually supersede one another [1,2]. Two of the major findings of next generation sequencing studies are that biopsies taken at the time of tumor presentation already contain a significant amount of genetic heterogeneity [5,6] and importantly that often a rare subclone in the primary tumor subsequently becomes the founding clone of a metastatic or relapse tumor [5,6,9,10]. The remarkable accuracy and read coverage depth of whole genome sequencing technology now enables the inference of intra-tumor heterogeneity and cancer evolutionary history that is encrypted in its mutational profile [2,9,11–14]. The study of the evolutionary history of a cancer provides insight into which mutations are cancer-driving from the numerous passenger mutations and sheds light on the mechanism of tumorigenesis.

Rhabdomyosarcoma (RMS) is the most common soft-tissue sarcoma of childhood. Despite a growing understanding of the molecular mechanism underlying RMS, the disease continues to have significant mortality and morbidity especially when the tumor is metastatic or recurrent [15–17]. RMS tumors can be subdivided into two major subtypes: PAX fusion positive (PFP) and fusion negative (PFN), characterized by the presence or absence of a oncogenic fusion between the *PAX3* or *PAX7* and *FOXO1* genes [18]. Fusion positive tumors tend to occur in adolescence and are associated with an adverse outcome. Fusion negative tumors typically occur at a younger age and have been associated with significant aneuploidy, loss of heterozygosity (LOH) at chromosome 11p15.5 [19] and mutations of *NRAS*, *KRAS*, *HRAS* [20], *PIK3CA*, *CTNNB1* [21,22] and *FGFR4* [22]. We have recently reported that PFP- and PFN-RMS have distinct landscapes of somatic genomic alterations that activate a common molecular pathway [23]. Another effort, using high coverage targeted re-sequencing of two relapsed fusion negative tumors, showed that indeed RMS relapse tumors are derived from a minor subclone discovered in the primary tumor [24].

In this study, we decipher for the first time, the evolutionary history of RMS using high coverage whole-genome sequencing of 44 primary tumors with their matched normal samples. We applied a framework of algorithms that enabled the prediction of the sequential order of mutational events and subclonality using chronological molecular information encoded in somatic mutations and allelic copy number (including copy number changes and allelic imbalance). The major inputs to our method include the variant allele fraction (VAF) of somatic mutations, the variant allele fraction of germline single nucleotide variants in tumor samples and somatic copy number status. We verified the accuracy of mutation detection and VAF estimation by deep sequencing on independent platforms. Our framework first estimates the rate of normal cell contamination and corrects its effect on VAF and copy number. Tumor subclones with different genomic profiles are then identified using the distribution of VAF of somatic mutations as well as the allelic copy number status. Finally, we estimate the temporal progression of the observed somatic events in each tumor genome based on the fact that somatic mutations that occur before and after aneuploidy events will have different values of VAF.

Through our analysis, we discovered that the initiating common lesions of PFN-RMS are the combination of loss of heterozygosity of chromosome 11p15.5 and point mutations in members of the RAS pathway in majority of cases. In a small number of PFN-RMS tumors where no obvious RAS pathway mutations were present, we identified alternative genes that were mutated early in the tumors progression, indicating their potential roles in oncogenesis. In PFP-RMS, we discover that a whole genome duplication event which results in tetraploidy consistently occurs in the middle or late stage of the development of these tumors and that the PAX3-FOXO1 fusion event occurs prior to the whole genome duplication event, making it probable that the fusion is an early event in the evolutionary history of these fusion-positive RMS. Finally, we find that in general RMS tumors are universally composed of a dominant clone, although each tumor contains subclonal populations with a unique mutational profile, which may provide selective advantage to a relapse or metastatic tumor. Because we sequenced only one tumor sample for each patient, a limitation of our study, despite a moderately high coverage of the whole genome sequencing (average 105X), is that we may miss some subclonal mutations and clones especially for subclonal mutations with a VAF<0.1. Nevertheless our findings allow us to propose a developmental model of how this devastating pediatric cancer initiates and evolves prior to presentation.

## Results

### Large-scale deep WGS analysis of RMS

We used data from whole-genome sequencing (WGS) of 44 primary RMS tumors (19 PAX- fusion-positive, and 25 PFN tumors) with paired blood samples (providing germline status) with an average of 105x coverage per genome base. The accuracy of somatic mutation detection has been estimated as 93% by experiments in which 604 non-silent mutations were verified by whole-exome sequencing and targeted sequencing on independent platforms [23]. To verify the observed VAF of somatic mutations, we re-sequenced the somatic mutations discovered in two RMS samples using multiplex PCR and deep sequencing (1997x coverage) with an orthologous platform. The verification rate of VAF was high by the targeted sequencing (90% accuracy respectively, S1 Fig.). In order to accurately estimate the timing of somatic alterations and dissect intra-tumor subclonality, we first estimated the portion of normal cells contaminating the tumor sample by surveying allelic copy number status and VAF of somatic mutations across the genome (S1 Text). The normal cell contamination rate was generally low in the 44 tumor samples, with a range of 0–33%, a median of 16% and a standard deviation of 9% (S1 Table). The effect of normal cell contamination was numerically corrected for in all subsequent analyses so that the VAF and copy number status are purely for tumor population. The efficacy of normal cell contamination correction is illustrated in S2–S4 Figs., where the observed non-integer allelic copy number was corrected to integers and the observed VAF distribution of somatic mutations was corrected to the expected distribution, *e*.*g*. VAF = 0.5 for heterozygous mutations on chromosomes without aneuploidy.

### Subclonality analysis

Multiple studies have shown that subclones exist in individuals with cancer which may become a major clone at relapse or progression and this phenomenon has been reported in two fusion negative RMS tumors [24]. To identify those events which may provide resistance to therapy and allow recurrence, we searched for genomic alterations that specifically were confined to the subclonal population. Subclonal copy number alteration events and mutational events were identified using an *in-silico* method (for which the workflow is shown in S5 Fig.). Fifty point mutations were identified where the variant’s allele frequency was greater than 10% from the expected full clonal allele frequency (S2 Table). Included in this gene list were 7 COSMIC genes including *ABL1*, *BUB1B*, *CDK12*, *ERBB2*, *IGF2*, *KDR*, and *SMARCA4*. Gene ontology showed enrichment for genes involved in cell adhesion (GO:0007155 p = 0.002), negative regulation DNA repair (GO:0006281, p = 0.05) and cell migration (GO:0030335 p = 0.026). Across the population, several chromosomal events were recurrently found in the subclonal population, including gain of chromosomes, 5, 8, 11, 13, 14, 18 and 19 (S1 Table).

**Fig 1:**
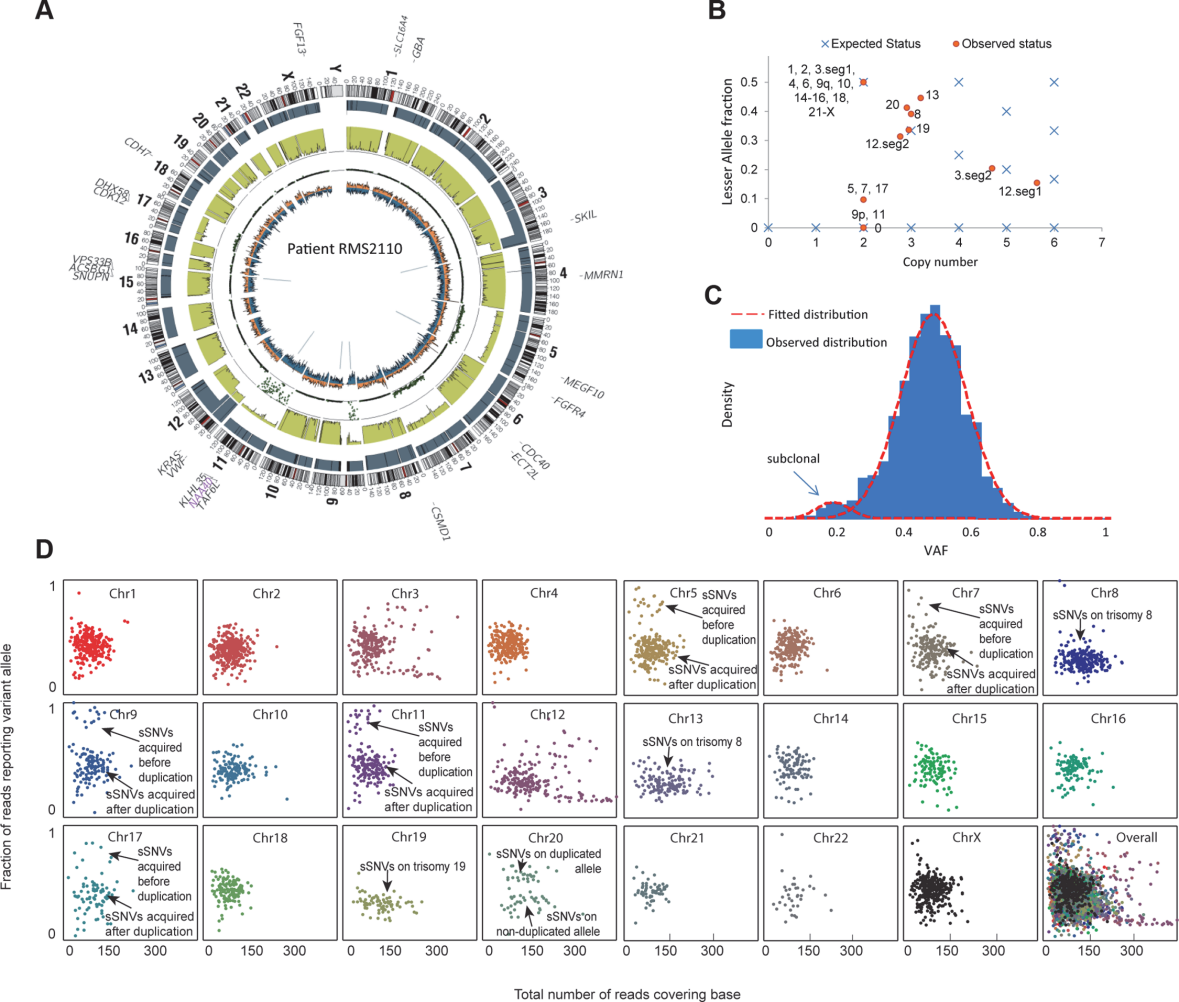
Inferring evolutionary history for a typical rhabdomyosarcoma sample RMS2110. (a) A circos plot of tumor genome RMS2110 (before normal cell contamination correction). Gene symbols indicate genes with nonsynonymous mutations. Tracks from outermost to innermost depict chromosome banding, copy number (the height of bars denotes copy number), lesser allele fraction (the height of green bars represents the fraction of lesser allele at each genomic location, valued 0∼0.5), loss of heterogeneity status (each dot represents the probability of loss of heterozygosity for the adjacent segment), intensity of heterozygous (orange bar) and homozygous single nucleotide variants (blue bar), junctions or chromosomal rearrangement (grey lines). (b) Discrepancy between expected and observed status of allelic copy number suggests the existence of subclone(s) in tumor. Red dots denote chromosome segments, whereas blue crosses denote statuses of copy number and lesser allele fraction that leads to an integer allelic copy number. If there are tumor subclones with copy number status different from the major clone, the red dots will deviate from the blue crosses. (c) Observed VAF distribution of somatic mutations on chromosomes without aneuploidy. The normal-mixture like distribution suggest the existence of a minor subclone with VAF<0.5. (d) A scatter plot showing coverage (horizontal axis) and VAF (vertical axis) for somatic mutations (sSNV) on each chromosome.

We illustrate our method in more detail using one PAX-fusion negative RMS tumor (RMS2110). Genome wide, this tumor had 3,889 somatic mutations including oncogenic mutation *KRAS* G13D and *FGFR4* V550L [23]. Using the procedure described in the previous section, we estimated that this sample had 12% normal cell contamination (S1 Table). Copy number analysis showed that RMS2110 had large-segment allelic imbalance (including copy neutral LOH) and copy number alteration on eleven chromosomes (Fig. 1A).We employed the combination of somatic mutations, allelic imbalance and copy number alterations to dissect tumor clones that have unique genomic profiles. First, VAF of somatic mutations was determined to find subclonal mutations, based on the fact that a subclonal mutation (present only in a part of the tumor cells) usually has lower VAF than full-clonal mutations (present in all tumor cells) [2]. A typical scenario is on chromosomes without aneuploidy or allelic imbalance, where heterozygous full-clonal mutations have VAF equal to 0.5 while subclonal mutations have VAF<0.5. Analysis of RMS2110 showed that more than half of the chromosomes are without aneuploidy or allelic imbalance (Fig. 1A and 1B). On these chromosomes, a small portion of somatic mutations have observed VAF significantly lower than others indicating the presence of subclones (Fig. 1C, where there are two distinct VAF clusters, one is centered on VAF = 0.5 and the other is centered on VAF = 0.2; for individual chromosomes see Fig. 1D). Using a clustering algorithm with cluster-number-selection procedure (S1 Text) we can identify subclonal mutations as those with lower VAF. Given the depth of sequencing coverage, we estimated that we could detect subclonal mutations with VAF as low as 0.1 (S6 Fig.). Second, allelic copy number status is used to detect subclonal copy number alterations, based on the fact that subclones of different copy number will result in a non-integer allelic copy number for the whole tumor sample. The joint status of total copy number and lesser allele fraction (LAF—the ratio between the less allelic copy number and total copy number, estimated by germline single nucleotide variants, see Methods) reflects whether the allelic copy number is an integer—whether the observed allelic copy number (red dots) is on the expected position (blue crosses) in Fig. 1D. Therefore our approach predicts the subclonal copy number alterations and the fraction of tumor cells possessing these changes (S1 Text).

We thus predict that the subclonal somatic mutations made up only 5% of all the somatic mutations in RMS2110 tumor. Subclonal copy number alterations were detected on chromosomes 5, 7, 8, 12, 13, 17 and 20.

**Fig 2:**
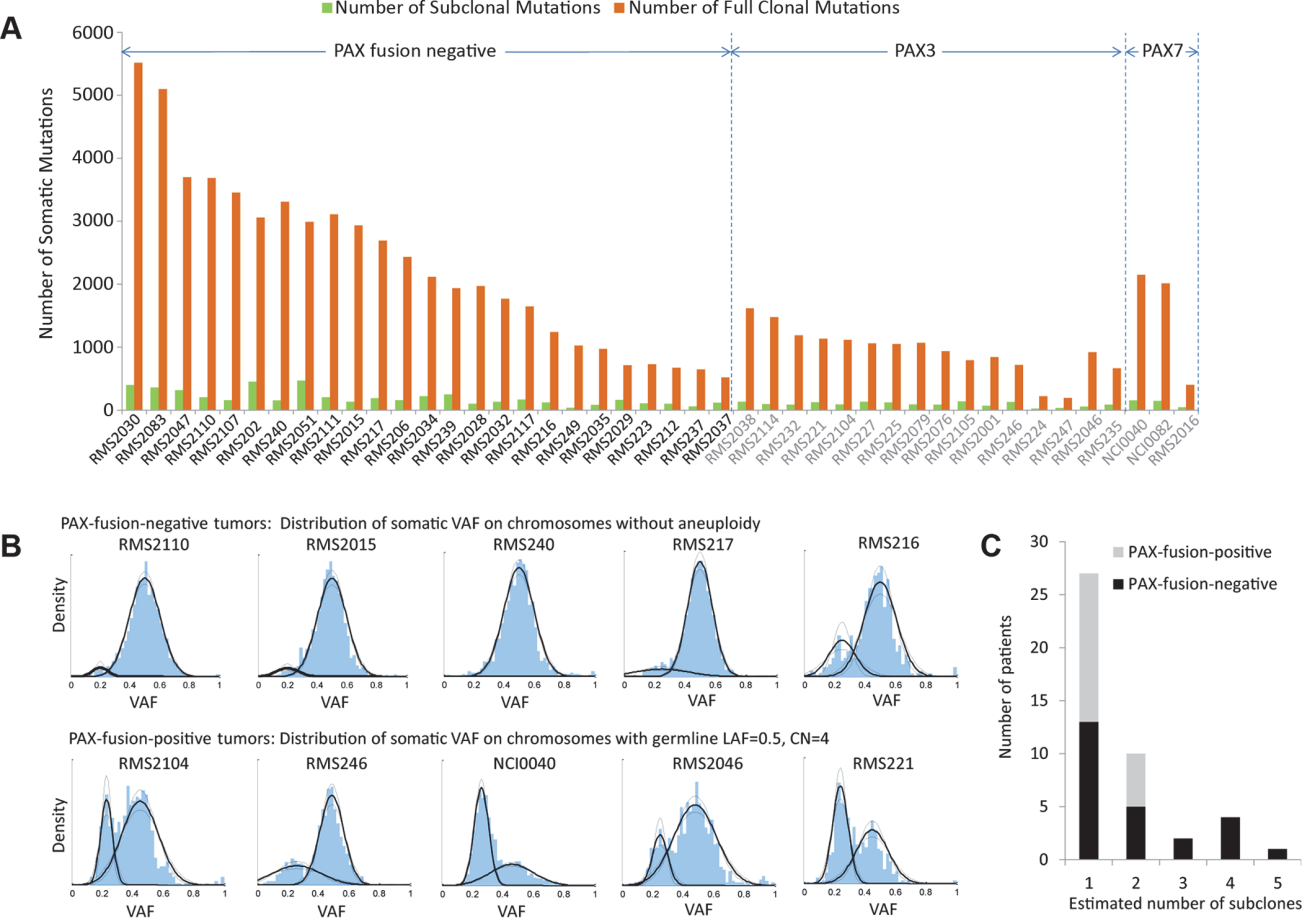
Subclonality in in 44 rhabdomyosarcoma samples. (a) Only a small number of somatic mutations are subclonal while the majority of them were present in all tumor cells. (b) The top panel shows the distribution of VAF of somatic mutations on chromosomes without aneuploidy for selected PFN-RMS samples, where a dominant clone is accompanied with a subclone(s). The lower panel shows the distribution of VAF of somatic mutations for selected PFP-RMS samples. Because these samples have undergone whole-genome duplication, the VAF of somatic mutations on tetraploid chromosomes is distributed around 0.5 and 0.25. (c) PFN samples have more detectable subclonal copy number alterations than PFP samples (p = 0.05, from Mann-Whitney test). It is possible that there are additional subclones that possess subclonal mutations or copy number alterations undetectable with our sequencing depth.

The method was then applied to all the 44 RMS tumors. Looking across all 44 genomes revealed that a dominant clonal lineage was present in each tumor sample (Fig. 2A). The dominant clone carried a large proportion (from 81–96%) of somatic mutations regardless of PAX-fusion status of the tumor. Meanwhile, a small percentage of the cells in each tumor did display evidence of subclonal changes (Fig. 2B). In addition, we observed more subclonal aneuploidy events in PFN tumors (more than half of the tumors have detectable subclones) than PFP tumors (Fig. 2C). The inferred subclonal aneuploidy and mutational events were listed in S1 and S2 Tables, respectively.

### Inference of the RMS cancer evolutionary history

The timing of genomic alteration events can be derived using chronological molecular information encoded in the somatic mutations and copy number alterations, because a specific order of genomic event will result in different VAF [2]. To explain the workflow of our method, we again take sample RMS2110 as an example.

**Fig 3:**
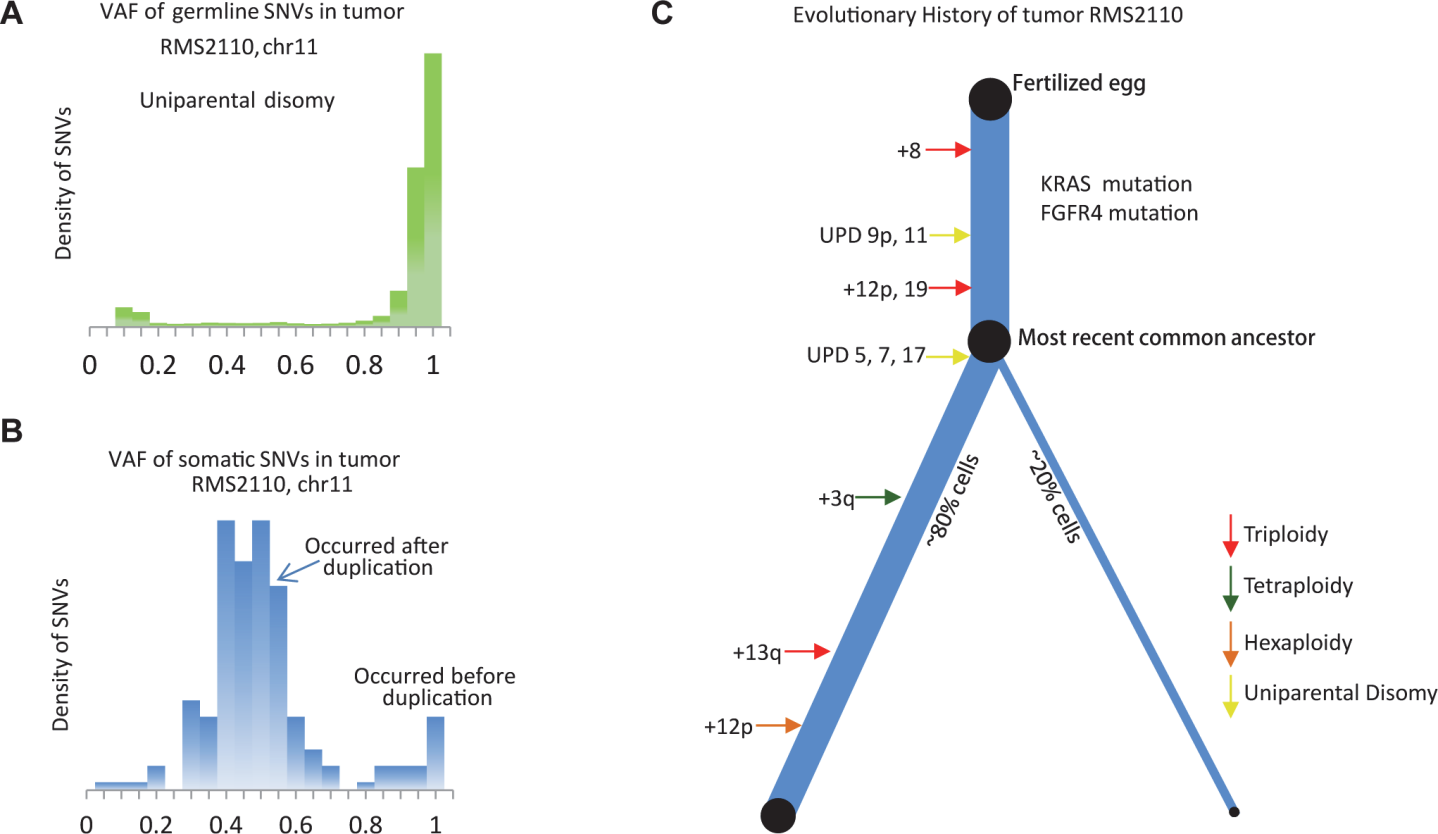
Inferring evolutionary history of a PFN sample, RMS2110. (a) The VAF of germline SNVs on chromosome 11 is distributed around 1, as a result of the uniparental disomy (LOH with duplication). (b) The VAF of somatic mutations is quite different from that of germline SNVs, suggesting majority of somatic mutations happen after the duplication event and thus are present in only one copy of chromosome 11. The earlier occurrence of the duplication of chromosome 11, the more somatic mutations with VAF = 0.5 are detected. S7 Fig. provides a more illustrative explanation. (c) Inferred evolutionary history represented as a phylogenetic tree. The thickness of the branches reflects the proportion of tumor cells in each lineage. The length of the branches reflects how much molecular time it undergoes with each lineage.

The majority of this cancer genome was the expected diploid status with LAF = 0.5, however regions with aneuploidy or allelic imbalance provided the opportunity to identify the timing of genomic events, by comparing the allelic copy number status with the VAF distribution of somatic mutations (S7 Fig.). For example, chromosome 9p and 11 have 2 copies with LAF of 0 (Fig. 1B), which is likely the loss of one allele followed by a duplication of the remaining allele although we cannot formally exclude the possibility of the duplication of both alleles with subsequent loss of the 2 copies of one allele. This observation was confirmed by the fact that most germline single nucleotide variants on chromosome 9p and 11 had VAF near 1 (Fig. 3A). In this case, the somatic mutations occurring before the “LOH+duplication” event must be present on both copies, with an expected VAF of 1, whereas those occurring after the “LOH+duplication” event would be present on only one copy, with an expected VAF of 0.5. The data confirmed this prediction with the VAF displaying a bi-modal distribution with two peaks at 0.5 and 1, respectively (Fig. 3B). The ratio between the numbers of mutations in the two clusters reflects the fraction of “molecular time” it undergoes to accumulate mutations before and after the “LOH+duplication” event, assuming a constant accumulation rate [2]. We acknowledge that the somatic mutation accumulation rate varies among small genomic segments [5,25–27], but for chromosome-level segments used in this study, the average accumulation rates of somatic mutation were observed to be consistent with one another (*r* ^2^ > 0.98, S8 Fig.). Therefore, the molecular timing inferred for different aneuploidy events were comparable among the segments within the same tumor sample.

This analysis was extended genome-wide and allowed the inference of the "phylogenetic tree" of tumor RMS2110 (Fig. 3C). Somatic mutations occurring before and after chromosome aneuploidy events had distinct VAF values, which allowed inference of the timing of two candidate driver mutations in this tumor, the nonsynonymous mutation of *FGFR4* V550L and the nonsynonymous mutation of *KRAS* codon G12. The mutation of *FGFR4* V550L has a VAF = 0.95, indicating that the mutation happened before the uniparental disomy event of chromosome 5, which is estimated to occur at 26% of molecular cancer lifetime (S1 Table). Therefore *FGFR4* mutation should happen between 0 to 26% of molecular time. Similarly, the mutation of *KRAS* codon G12 has a VAF of 1, indicating that it occurred before the copy gain of chromosome 12p, timed at 21% of molecular cancer lifetime. Other early events included trisomy of chromosome 8 which happened at 8% of the molecular cancer lifetime and uniparental disomy of chromosomes 11 and 9p which occurred at 14% of the molecular cancer lifetime. Note that on both copies of chromosome 9p, there is a somatic focal deletion of *CDKN2A* (chr9:21981721-21952010), which is also verified by RNAseq (FPKM = 0).

Other aneuploidy events in this sample included: trisomy of chromosomes 12 and 19 which occurred at 21% of molecular cancer lifetime; uniparental disomy of chromosomes 5, 7 and 17 which happened at 26% of molecular cancer lifetime; tetrasomy of a segment of chromosome 3q which occurred around 50% of molecular cancer lifetime; trisomy of chromosome 13 which occurred at 77% of molecular cancer lifetime; hexasomy of 12p which happened at 85% of molecular cancer lifetime; and trisomy of chromosome 20 which occurred near the time of tumor presentation (Fig. 3C).

**Fig 4:**
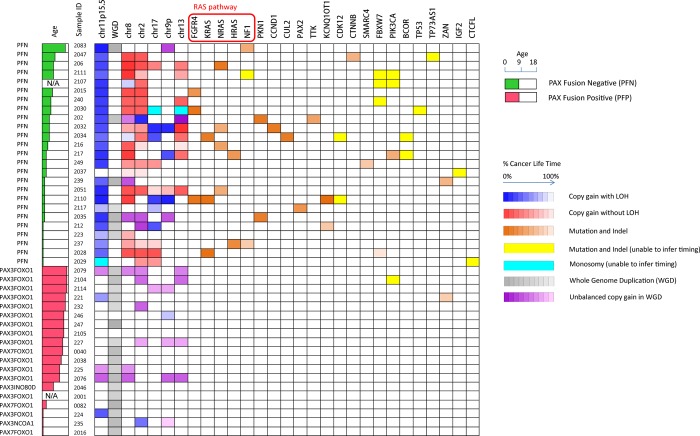
Summary of recurrent somatic lesions found across the 44 tumors and the estimates of their occurrence time. The heatmap denotes the occurrence of the lesions and their timing (the brightness of grids represents the percentage of cancer lifetime when the lesion occurred). The occurrence time of a mutation is represented as the median of its inferred time interval. Note that within a single chromosome, there may be multiple segments with different copy number status, and we only show the arm-level copy number alterations in this figure. 11p15.5 LOH with copy gain and the mutations in RAS pathway are consistently early events during the development of PFN rhabdomyosarcoma.

This method was applied to all the 44 tumor-normal sample pairs to build the evolutionary history of RMS (Fig. 4, S9 Fig., and S1 Table). In summary, our results showed three major findings. First, LOH of 11p15.5 was a consistent early founding event (in average occurred at 35% of molecular cancer lifetime) in PFN-RMS. In comparison, other highly recurrent aneuploidy events such as the copy gain of chromosome 8 and 2 were not consistent early occurring events (time ranges from 1%∼95% and 16%∼96%, respectively). Second, mutations in RAS pathway genes, including *FGFR4*, *KRAS*, *NRAS* and *HRAS*, were recurrent early events in PFN-RMS. In addition, mutations in other genes (*PKN1*, *CCND1*, *CUL2*, and *TTK*) occurred early suggesting their role in tumorigenesis. Third, PFP-RMS tumors in general had much fewer somatic alterations and few of them occurred early in the tumor’s molecular lifetime. Of note, a whole-genome duplication event consistently occurred at the middle or late point in the molecular lifetime of these tumors. The high recurrence suggests that this event might be crucial for the presentation of this cancer subtype. We will discuss these findings in more details in the following sections.

### Loss of Heterozygosity of 11p15.5 is a common founding event in PAX fusion negative RMS

The LOH of 11p15.5 is a critical event in fusion-negative tumors [19,28] and frequently results from uniparental disomy and trisomy. In our study, the LOH of 11p15.5 occurred in a total of 26 RMS tumors. As previously described, there was a distinct enrichment for the event in the PFN-RMS population (24/25 PFN samples). Of note, the remaining PFN sample had a small deletion event (3 bps) in the 3-prime non-coding region of *IGF2* of undetermined significance. In contrast, only 2 out of 19 PFP-RMS samples had 11p15.5 LOH. The observed LOH usually was accompanied by a copy gain (23 out of 24 PFN samples with LOH on 11p15.5) of the remaining allele resulting in uniparental disomy (n = 8 in PFN and n = 1 in PFP), trisomy (n = 4 in PFN), tetrasomy (n = 8 in PFN and n = 1 in PFP), or pentasomy (n = 3 PFN) (S10 Fig.).

Applying our evolution-history-inference method across the RMS population revealed that the 11p15.5 LOH event universally occurred at an early time point in these tumors development (S11 Fig.). In these tumors, 11p15.5 had a LAF of 0 (S12 Fig. (A)-(C)), meaning that most germline single nucleotide variants present in the tumor, were homozygous. In contrast, the somatic mutations can be homozygous (before 11p15.5 copy gain) or not (after 11p15.5 copy gain). For instance, on chromosome 11 (uniparental disomy) of sample RMS2110, the majority of somatic mutations distributed around a VAF of 0.5 with a minority distributed around VAF of 1 (Fig. 3B). The cluster of somatic mutations discovered around a VAF of 0.5 indicated that these mutations occurred after chromosomal duplication. Comparison of the relative size of the mutations at VAF of 0.5 to those at VAF of 1 indicated that the duplication of chromosome 11 happened early in the cancer evolutionary history. On average, the “LOH+duplication” event of 11p15.5 occurred at 35% of molecular time of the 23 PFN-RMS tumors that had this event. The early occurrence of 11p15.5 “LOH+duplication” event was especially prominent in the patients diagnosed at a later age—the mean occurrence molecular time is 29% of lifetime and the variance is 10% in ≥3 year old patients (S1 Table).

In contrast to the high recurrence (92%) of 11p15.5 LOH in PFN tumors, 11p15.5 LOH was found in only 11% of our PFP tumors. Moreover, in contrast to the consistent early occurrence in PFN tumors, the LOH event did not consistently happen at early lifetime of PFP tumors—37% and 64% of lifetime for two PFP tumors, respectively.

Beyond 11p15.5, the LOH of chromosome 17 (6 out of 25 PFN-RMS) was identified as a recurrent alteration event, typically occurring at an early time point in the evolutionary history of RMS. The LOH region on chromosome 17 includes two small genomic regions that encompass the *TP53* and *NF1* locus, respectively (S10 Fig.). The observed lesions occurred at an average molecular timing of 38% of cancer evolutionary history. The LOH of 9p, was present in 3 out of 25 PFN-RMS and was found to occur at an average 41% of cancer evolutionary history. Additional accumulation of aneuploidy, such as gain of chromosome 2, 8 and 13, frequently followed the founding events and had no consistent timing pattern.

### Whole genome duplication in PAX fusion positive tumors

Unlike PFN-RMS tumors which have significant chromosomal rearrangements, PFP tumors typically have an LAF at the expected 0.5 and a consistent copy number status across the genome. The VAF distribution of somatic mutations revealed that many samples have genome-wide tetraploidy as evidenced by a cluster of somatic mutations with VAF around 0.25 (Fig. 2B, PFP tumors). In total, we found similar VAF distributions in 17 out of 19 PFP tumors. While it is possible that there were two distinct subclones each occupying 50% of tumor cells, given that the cluster with VAF around 0.25 is consistently found across multiple tumors, tetraploidy is a much more likely assumption. Interestingly, when we analyzed these 17 PFP cancer genomes chromosome by chromosome, we found that the inferred tetraploidy occurred in one apparent event (S13 Fig.). This event typically occurred around the mid-point of the molecular lifetime of the tumor (62%±16%) (S1 Table).

In order to identify the sequential order of the PAX3-FOXO1 fusion event and the whole genome duplication event we interrogated the number of the sequencing reads across the PAX3-FOXO1 junction and found that the junction is duplicated. In contrary to PAX7-FOXO1, PAX3-FOXO1 is not known to be focally amplified [29], thus the observed duplication of PAX3-FOXO1 is likely due to the whole genome duplication. Our analysis demonstrated that the PAX3-FOXO1 fusion was consistently duplicated in the whole genome duplication and thus was likely to have occurred prior to the whole genome duplication event (S4B Fig., S3 Table).

### Timing the evolutionary history of 44 RMS tumors demonstrates that RAS pathway mutations are critical events in the development of fusion negative RMS

In an effort to obtain a comprehensive picture of the evolutionary history of the RMS tumor genomes, we applied our method to estimate the timing of all the aneuploidy events and mutational events that occurred prior to each tumor’s presentation (Fig. 4). In addition to the recurrent and early 11p LOH event in fusion negative tumors, mutation of *FGFR4*, *KRAS*, *NRAS* and *HRAS* frequently occurred at an early time point in the cancers evolutionary history (S1 Table). Interestingly, many of the early-mutated genes belong to the RAS pathway (Fig. 4). In total 13/25 fusion negative samples carried the combination of early loss of LOH of 11p15.5 and the mutation of *RAS*, *NF1* or *FGFR4* which form the founding events in the evolutionary history of these tumors.

Timing and expression analysis of all the observed somatic mutations was used to discover potential driver mutations that occurred early during tumor development (S4 Table). These mutations included recurrent alteration in two PFN RMS of *PKN1* (<30% and <42% of molecular lifetime) which encodes a kinase belonging to the protein kinase C superfamily, *CCDN1* at <33% of molecular lifetime in RMS2032, *CUL2* at <33% in RMS2034, *PAX2* at <77% in RMS 2117, *TTK* at 17–78% in RMS202.

### The E216K mutation of PKN1 inhibits terminal differentiation of C2C12 cells

**Fig 5:**
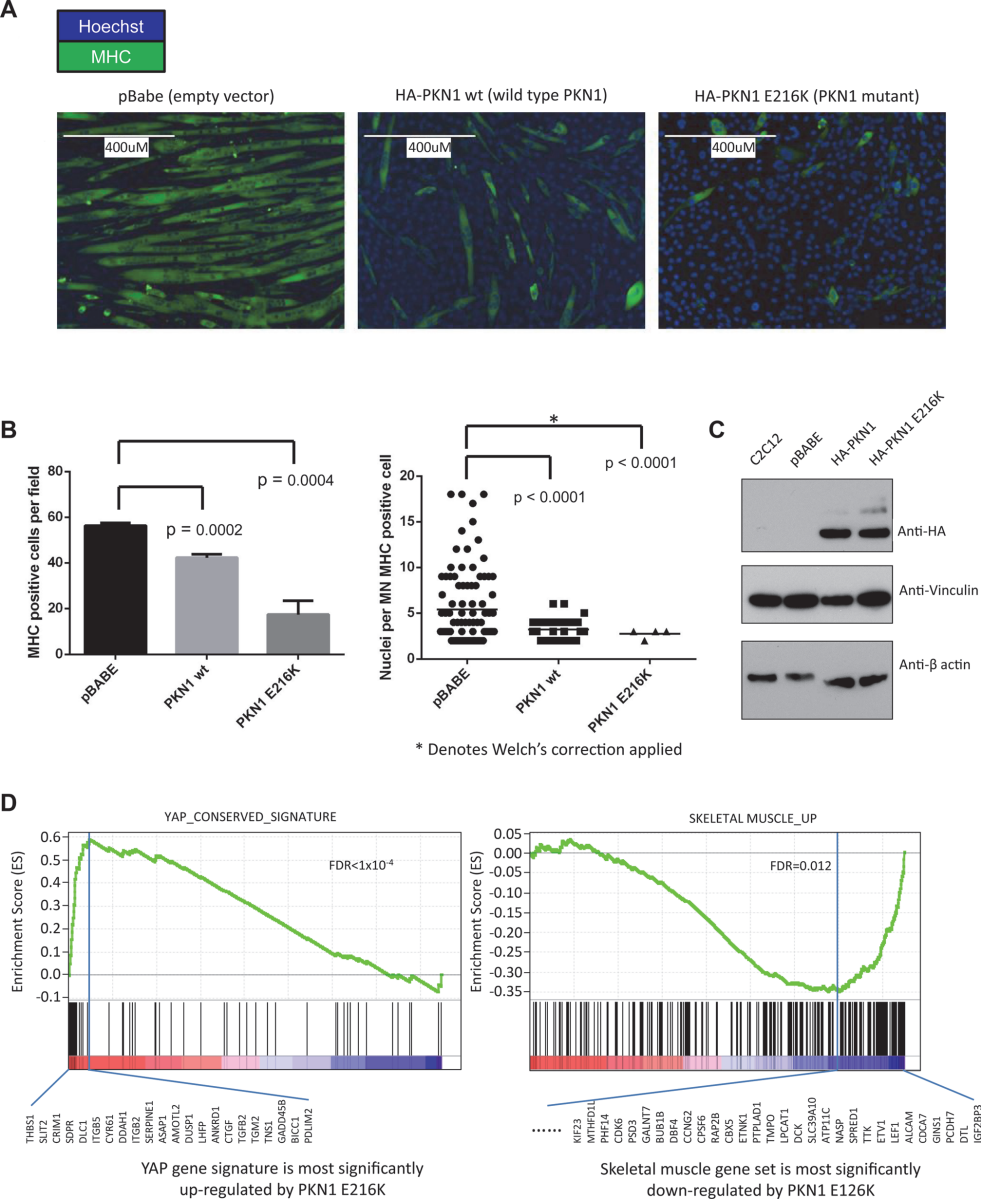
PKN1 E216K mutation prevented muscle differentiation in C2C12 mouse myoblasts. (a) C2C12 cells were transduced with viral constructs containing either empty vector (pBabe), wild type PKN1 (HA-PKN1 wt) or PKN1 E216 mutant (HA-PKN1 E216K). Immunofluorescence performed for myosin heavy chain showed a striking arrest of terminal muscle differentiation. The size bar represents 400um. (b) Quantitation of the observed phenotype showed a significant decrease myosin heavy chain positive cells when PKN1 E216K is expressed. (c) Immunoblot demonstrating expression of HA tagged wild type and mutant PKN1. (d) Gene set enrichment analysis (GSEA) of gene expression profiles demonstrated that PKN1 E216K mutant activated YAP1 target genes as well as cancer invasion-associated genes; while repressed muscle differentiation program in the myoblasts.

Recurrent and early mutation of *PKN1* in two of the evaluated tumors led us to hypothesize that these changes were involved in myogenic differentiation. To test our hypothesis and assess the potential of our method in identifying driver mutations, we conducted a cell line study described in this section. *PKN1* is a member of the AGC-subfamily of serine/threonine kinases. The protein product of the *PKN1* gene is composed of a C-terminal kinase domain with significant homology to that of the protein kinase C isoforms, but a unique auto-inhibitory N-terminus made up of 3 homologous stretches of anti-parallel coiled-coil folds (ACC1–3) known to bind to Rho-family GTPases in a nucleotide-dependent manner followed by a C2-like region, known to bind phospholipids and fatty acids. Rho-GTPase binding to the ACC regions causes a conformational change that allows *PKN1* to be phosphorylated and activated by PDK1. Active PKN1 plays a role in diverse cellular processes such as regulation of the actin cytoskeleton, cell adhesion, vesicular transport and glucose metabolism [30]. PKN1 represses WNT/CTNNB1 signaling [31] and stimulates the ATF2 and MEF2A transcription factors via a signaling pathway that involves MAP2K3/MAP2K6 and MAPK12 [32]. The observed mutations in RMS202 (E216K) and RMS2035 (A298T) occurred prior to 30% and 42% of molecular lifetime of the tumors, respectively. Both mutations occur in the region of the third ACC domain and could potentially interfere with regulation of the kinase activity of PKN1. To test the functional consequences of the PKN1 E216K mutation, wild type and mutant *PKN1* viral constructs were made and transduced into the mouse skeletal muscle precursor cell line C2C12. Using a differentiation assay described by [33], defects in terminal differentiation, reflected in expression of myosin heavy chain (MHC) were observed with the mutated version of *PKN1*, (Fig. 5A-C), suggesting that the mutant *PKN1* prevented the C2C12 cell differentiation. C2C12 cells expressing wild type *PKN1* could be induced to express MHC although cell fusion, as determined by the number of nuclei per MHC positive cell, was significantly inhibited. Since *PKN1* is known to regulate the activity of several transcription factors known to play a role in myogenic differentiation, we performed expression analysis of the constructed cell lines to determined differentially expressed genes among the constructs. Interestingly, gene set enrichment analysis showed that *YAP1* target genes were induced in myoblasts and skeletal muscle genes were repressed in myotubes (Fig. 5D) when the *PKN1* mutation was present.

## Discussion

While a growing number of studies have used next generation sequencing to examine the evolutionary development of adult tumors [2,9,13], this study is the first application of whole-genome sequencing to the study of a pediatric solid tumor’s subclonality and evolutionary history. The implication from adult studies is that progression of a tumor cell follows a long course, with subsequent lineages of cells acquiring mutations until a particular alteration allows expansion of one clone; ultimately leading to clinical detection. Studies of multiple cancer types including melanoma [34] and colon [35] have shown that even potent oncogenic mutations may be relatively quiescent and thus remain in an undetected premalignant state until released by an additional genetic hit. The patients in our study had a median age at diagnosis of 6.5 years, indicating that the tumors in these children were developed over a relatively short time course, perhaps hinting at the relative potency of the observed somatic changes. Despite a shorter window of development, our study shows that the basic pattern of sequential mutation accumulation is maintained.

Given that similar analyses of adult tumors find the presence of a dominant clone [2], we anticipated that RMS tumors might have a similar finding. Our results show that a dominant clone accounts for more than 80% of cancer cells in a typical tumor sample and genome wide carries many hundreds or even thousands of accumulated point mutations. Even with our genome-wide sequencing and deep coverage, the subclones are frequently observed only at the limits of detection. Admittedly, our study may miss part of the subclonal mutations due to the limit of coverage and the complex nature of genomic subclonality, especially for subclonal mutations with a VAF<0.1. Nevertheless GO analysis of subclonal mutations showed enrichment for potential resistance and progression pathways including cell adhesion, DNA repair and cell migration. While our DNA extraction included relatively large tumor sections, it is also possible that further sampling of the tumor may yield additional subclonal populations as have been appreciated in the study of distant metastases [10]. Even with these constraints, we can conclude that one clone typically dominates these samples, and the majority of tumor cells share most of the detectable genomic alterations. In addition, our findings indicate that a significant proportion of the clonal heterogeneity in RMS is found in gain or loss of copy number, mirroring similar findings in a study of pediatric acute lymphoblastic leukemia [36]. Future efforts will be needed to determine if these alterations might play a role in defining a "cancer stem cell" which resists the selective pressures of the microenvironment and therapy to survive as a recurrence or metastatic lesion.

**Fig 6:**
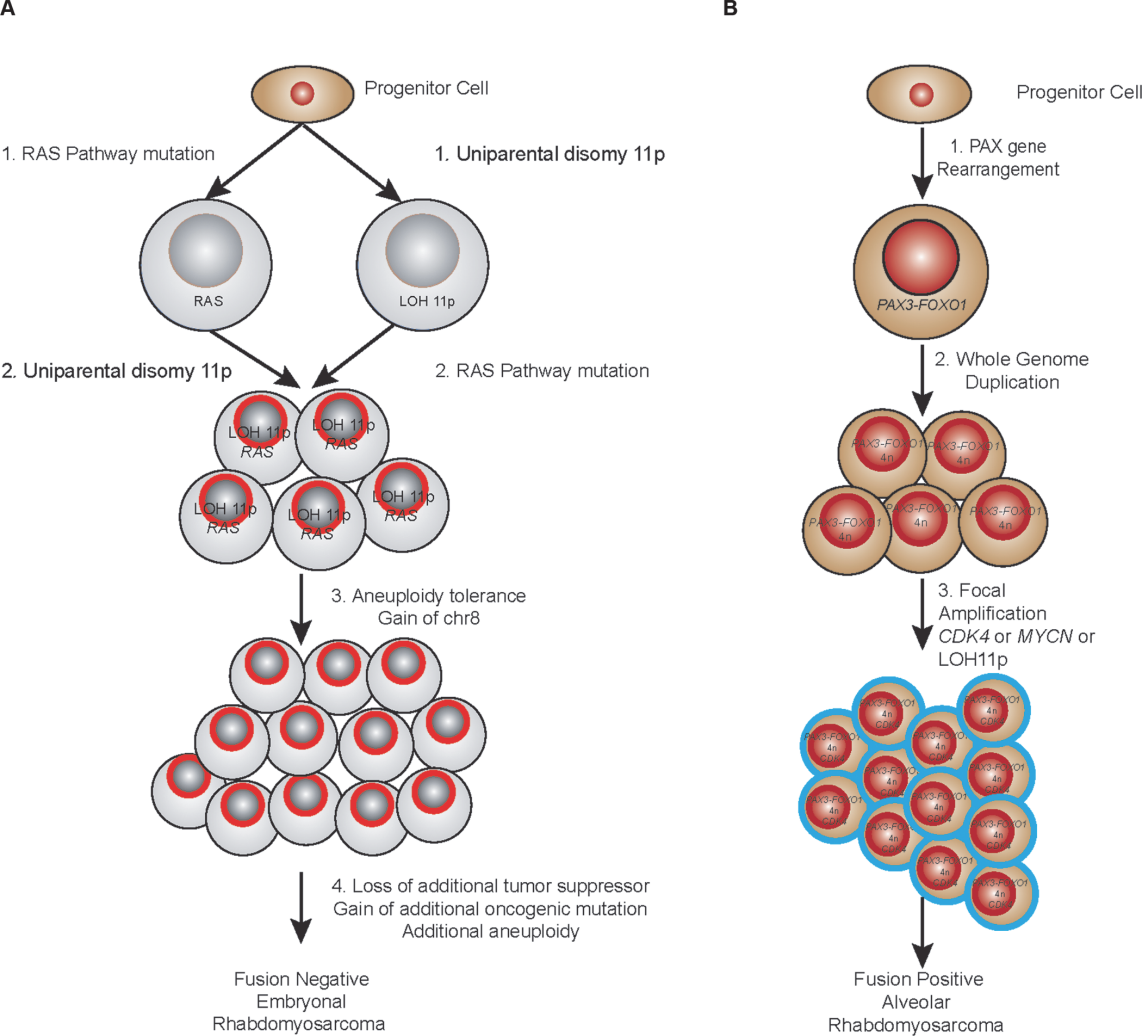
Proposed evolutionary model of rhabdomyosarcoma. (a) PFN samples. (b) PFP samples.

From the analyses described here, we can begin to understand the dynamics of RMS development. LOH of 11p15.5 has long been described as recurrent feature of several pediatric conditions including the overgrowth phenotype of Beckwith-Wiedemann Syndrome [37], Wilms tumor [38] and embryonal RMS. The proposed mechanism for oncogenesis of this lesion is loss of imprinting control over the *IGF2* locus resulting in over-expression of this developmentally regulated growth factor. In this study, we find that not only is this lesion highly recurrent (>90%), it also appears to be the key early landmark in the evolution of fusion-negative tumors. The discovery of a somatic mutation of IGF2 within a fusion negative sample that does not harbor LOH of 11p15.5 (RMS2037) provides additional support to the role of dysregulation of IGF2 in PFN-RMS. In evolutionary terms, the presence of 11p15.5 LOH defines the “most recent common ancestor” when combined with a mutation in a gene in the RAS pathway (*NRAS*, *KRAS*, *HRAS*, *FGFR4*). While the progression we describe in this study indicates a possible common sequence of events (S1 Table), in some tumors it is equally as likely that the oncogenic mutation of a RAS pathway gene is the founding lesion. This corroborates the finding that patient’s with Costello Syndrome (*HRAS* germline mutation) [39], Noonan Syndrome (*NRAS*, *KRAS*, *PTPN11* germline mutations) [40] and Neurofibromatosis (*NF1* germline mutation) [41] all have increased risk of developing fusion-negative RMS. Interestingly, while RMS is certainly described in patients with Beckwith-Wiedemann Syndrome (germline uniparental disomy of 11p15.5), these patients appear to have a higher relative risk of developing Wilms’ tumor and hepatoblastoma than RMS [42]. Regardless of which lesion comes first, the combination of LOH of 11p15.5 with a RAS pathway mutation appears to set a clone on the course towards developing a fusion negative RMS tumor (Fig. 6A).

By examining other mutations that occur early in the molecular history of fusion negative tumors, especially those tumors that had no mutation in a candidate “RAS” pathway member, it is possible to nominate other potential founding lesions. In this analysis, mutation of *PKN1* stands out as both recurrent (occurring in tumor RMS202 and RMS2035) and occurring early in the evolutionary history of these tumors (<30% and <42% respectively). PKN1 is a member of the protein kinase C family and has been implicated as a repressor of WNT/CTNNB1 signaling [43], a mediator of insulin signaling to the actin cytoskeleton [44] and an activator of MEF2A dependent transcription [32]. The observed mutations (E216K and A298T) occurred within the third ACC domain and may relieve N-terminal auto-inhibition of PKN1 kinase activity (S14 Fig.). Our functional assessments of the *PKN1* mutation demonstrated that the observed mutation can inhibit terminal differentiation of skeletal muscle in a dominant-negative fashion. Gene expression analysis of PKN1 E216K expressing C2C12 cells grown in low serum media reveals that this differentiation block occurs as a result of repression of skeletal muscle specific genes and genes with MEF2A binding sites in the promoter. Expression of MEF2 proteins is induced by myogenin during normal skeletal muscle differentiation, and MEF2 factors, in complex with other transcriptional activators, are known to play a role of in myogenic differentiation [45]. One possible explanation for these results is that PKN1-dependent signaling leads to replacement of MEF2A at skeletal muscle specific genes with a transcriptional repressor. The identity of this transcriptional repressor is not yet known.

GSEA analysis of PKN1 E216K expressing C2C12 myoblasts reveals that the gene expression signature of these cells is enriched for a YAP gene signature. The YAP gene signature is composed of genes activated by YAP overexpression in human mammary cells, mouse liver tissues and immortalized mouse fibroblasts, and thus represents a list of evolutionary YAP target genes [46]. YAP1 has recently been identified as a potent ERMS oncogenic driver, and YAP1-TEAD complexes repress expression of genes important for myogenic differentiation, in part by impinging upon the binding of myogenic transcription factors including MEF2 [47]. Intriguingly, YAP1 is able to functionally compensate for oncogenic KRAS in colorectal and pancreatic cancer mouse models [48,49], which is consistent with the fact that RAS mutation and *PKN1* mutation are mutually exclusive in the set of ERMS tumors presented here. The mechanism by which PKN1 leads to increased YAP1 activity is currently unknown.

With whole genome sequencing from a single biopsy, we were unable to reliably estimate the molecular timing of some of the recurrent genetic lesions observed in RMS. These events include mutations in *PIK3CA*, *FBXW7*, *NF1*, and *BCOR*, and the focal amplification of *MDM2*, *CDK4*, and *MYCN*. Moreover, somatic events such as the copy gain of chromosomes 2, 8 and 13 were estimated to occur in an inconsistent temporal pattern among RMS patients (occurring at multiple time points throughout the evolutionary history). Certainly, the recurrence of these lesions in multiple large sample sets speaks to their importance, however since they can occur either early or late in the cancers evolutionary history they do not represent the initiating lesion in the tumor but rather modifiers of the tumor as it grows towards presentation.

Early flow cytometry studies to evaluate DNA content [50] and cytogenetic analysis [51] in ARMS noted the frequent presence of tetraploidy. We find evidence that fusion positive tumors demonstrate a very high recurrence of a whole genome duplication event that results in a tetraploid genome. In general, the duplication event occurs around the midpoint of the tumor’s evolutionary history and prior to additional accumulation of aneuploidy (Fig. 6B). The mechanism of a cell moving from a diploid state to a tetraploid state can be due to a cytokinesis failure, a cell fusion event or mitotic slippage and it has long been speculated that tetraploidy is an intermediate in cancer progression [52]. This is born out in at least one premalignant state (Barrett’s esophagus), where tetraploidy represents an early intermediate as the cancer precursor develops towards esophageal adenocarcinoma [53]. Further experimental evidence of a tetraploidy intermediate state is demonstrated in *TP53* null mouse mammary cells where tetraploid but not diploid cells promote tumorigenesis in nude mice [54]. Of note, in the mouse model of PFP ARMS both the increase in allelic copy number of the *PAX3-FOXO1* fusion and the loss of *TP53* or *CDKN2A* were found to be a critical component in increasing the penetrance of the phenotype [55]. Given the role of loss of *TP53* in allowing a permissive environment for the tetraploid cell to escape cell cycle arrest, this is consistent with our findings. It is interesting to speculate that in our tumor series the whole genome duplication event is an attempt by the tumor to increase the allelic dose of the PAX fusion. Another intriguing possibility is that the whole genome duplication event alters the telomere length and activates telomerase; a critical cooperating genetic event in the temporal sequence that produces alveolar rhabdomyosarcoma from human myoblasts [56].

In conclusion, based on deep whole genome sequencing, we developed a systematic method to infer the evolutionary history and identify the causative lesions of this pediatric solid tumor. From our data, we provide a model for how these tumors develop. Our results demonstrate two distinct evolutionary paths resulting in a convergent phenotype of this soft tissue cancer: 1) genomic loss of heterozygosity on 11p15.5, nonsynonymous mutations on RAS pathway and cell cycle genes, including *FGFR4*, *KRAS*, *NRAS*, *HRAS* and *CCDN1*, as well as several other genes, including *CUL2*, *TTK* and *PKN1*, occur early in the evolution history of PFN-RMS; 2) recurrent whole genome duplication occurs in the middle or late stage of the tumors evolution in the PAX-fusion positive RMS tumors. PAX3-FOXO1 fusion occurs before the whole genome duplication event. Intriguingly, a recent report of the clonal evolution in two cases of relapsed fusion-negative RMS tumors after chemotherapy and radiation demonstrated that recurrent tumors are derived from the founding tumor’s minor clone [24]. Nevertheless given that survival following relapse is 30% [57], our findings that LOH of 11p15.5 and mutation of RAS genes form the “trunk” of the fusion negative RMS tumors has important therapeutic implications; at least in theory it is preferable for treatment to target mutations that are present in all of the tumor cells. In addition, while further biologic validation is required, our analysis identifies candidate lesions that may underpin the ability of the minor clone to survive therapy and proliferate during relapse or metastasis. Thus given clonal evolution and heterogeneity, our data suggests that future efforts to understand the emergence of resistant clones should include deep sequencing analysis on all patients at presentation and relapse. These efforts will add to the growing understanding of the biology of RMS and identify actionable genetic aberrations for targeted therapies.

## Methods

### Patient samples

All patient sample collection was approved by the institutional review board of the participating facility. All de-linked and de-identified patient sample information was collected under the approved institutional review board of the National Cancer Institute protocol 10-C-0086. Samples were assembled from collections at the Pediatric Oncology Branch of the National Cancer Institute, Children’s Oncology Group, and the Tumor Bank at The Children’s Hospital at Westmead (New South Wales, Australia). All tumors were collected at initial diagnosis and prior to any therapy with the exception of samples NCI0040 and NCI0080 which were collected at relapse. Samples were de-identified and histologic diagnosis and clinical information were compiled. Quality control genotyping for the whole genome samples was performed to ensure the match of tumor normal pairs.

### Whole genome sequencing

Approximately 6 micrograms of DNA was sequenced using the paired end sequencing method of Complete Genomics. Data analysis was accomplished using CGA tools package v2.0 as well as a number of in house tools described in [23].

### Targeted resequencing

A custom panel of oligonucleotides designed to incorporate somatic single nucleotide variants discovered in samples RMS2110 and RMS2107 was generated using Ion Ampliseq designer software. 150 base pair amplicon libraries were generated using multiplex PCR according to the Ion Torrent Ampliseq Library 2.0 kit. Individual samples were barcoded and the generated libraries were sequenced using a 318 chip on a Personal Genome Machine (Life Technologies). Compiled reads were mapped to Hg19 and the expected variants were analyzed for coverage and VAF. The targeted sequencing has a depth of 1997x. We used it to verify the VAF estimates of somatic mutations across whole genome of two samples. The result shows VAF estimation accuracy is as high as 90% (S1 Fig.).

### SNP array

Illumina Omni 2.5M (97 paired plus 30 unmatched tumors) or 5M (10 paired samples) were performed according to the standard procedure from the manufacturer (Illumina, San Diego, CA) at the National Cancer, Cancer Genomics Research Laboratory. The data were previously reported in [23].

### Whole transcriptome sequencing

PolyA selected RNA libraries were prepared for RNA sequencing on Illumina HiSeq2000. 100 bases long paired-end reads were assessed for quality and reads were mapped using CASAVA (Illumina, San Diego, CA). The generated fastq files were analyzed by TopHat2 [58] and Cufflinks [59] The data were previously reported in [23].

### Somatic mutation calling

We used the somatic score from Complete Genomics (CG), a highly sensitive and specific somatic mutation-calling criteria [23,60,61], combined with principles previously used [25,62] to detect somatic signal nucleotide variants (SNVs). The somatic score is designed by systematically considering sequencing error, mapping error and read count and has been validated as an effective criterion by our previous studies as well as studies from other groups [23,61,63]. To ensure sensitivity and specificity of mutation calling, we set the somatic score cutoff as ≥0, with a set of additional filters for removing system artifacts and mapping errors, to select somatic mutations, based on the verification results from an independent sequencing platform [23]. By SOLiD whole exome sequencing (35x coverage) and Ion Torrent Ampliseq targeted sequencing (300x coverage) on 30 RMS tumor-normal sample pairs sequenced by CG, we verified that our filtering removes 99% of false positives while maintaining 80% of true positives [23] in CG’s comprehensive somatic mutation pool (called in a loose criterion to be as inclusive as possible) [23,60], which is premium given the less somatic mutations in pediatric tumors [3].

### Copy number profiling

For the 44 matched tumor and normal sample pairs with whole genome sequencing, we used somatic copy number segmentation profile provided by Complete Genomics, with customized corrections. For a larger cohort, with 120 matched tumor and normal sample pairs measured by SNP array, we used the copy number profile provided by NEXUS copy number analysis.

In copy number profiling, an important quantity, lesser allele fraction (LAF), is estimated to represent the fraction of copies coming from each parental allele. Along with total copy number, LAF tells us the allelic copy number status. LAF is defined as the ratio between the copy number of the lesser allele (with fewer copies than the other allele) versus the total copy number. For example, a trisomic chromosome with allelic type “AAB” has LAF equal to 1/3. LAF is estimated based on germline heterozygous single nucleotide variants that are present in cancer genome. For each genomic segment of tumor sample, sites that have heterozygous single nucleotide variants in matched normal samples were selected and LAF was estimated by the ratio between the read count of lesser allele and the total read count for these sites.

### Statistical analyses

In order to study the evolutionary history of RMS, we performed an integrated statistical analysis, using information including germline single-nucleotide variants, somatic mutations (single-nucleotide variants), somatic copy number alterations (CNA) and junctions (mapped breaking points), to estimate the normal cell contamination, intra-tumor heterogeneity (due to subclones) and the timing of somatic variants. The methods are detailed in the S1 Text.

### Cell lines and retroviral constructs

Mouse myoblast cell line C2C12 was obtained as a generous gift from Dr. Marc Landanyi. The cells genotype was performed by the NCI Core Genotyping facility and the cell line was confirmed to be myocoplasm negative. All cell culture was performed in DMEM supplemented with 10% FBS. The differentiation assay was performed using 2% horse serum (Life Technologies) as previously described [33]. For retrovirus production, the pBabe vector system (Addgene) was used.

### Retrovirus construction and transduction of C2C12 cells

Plasmids encoding human *PKN1 c*DNA was purchased from Addgene, and constructs were subcloned into pBABE containing a N-terminal HA tag. A cDNA encoding the PKN1 E216K mutant was generated using the GeneART Site-Directed Mutagenesis kit (Life Technologies) and was subcloned into pBabe. The generated mutation was confirmed by Sanger sequencing. Retroviruses were generated by contransfection of pBabe constructs with pCL-10A1 into 293T cells (American Type Culture Collection (ATCC), CRL-3216) subsequently used to infect the C2C12 cell line as previously described; infected cells were selected with 2 μg/ml puromycin (Life Technologies).

### Immunofluorescence

Cells grown on Nunc chamber slides were fixed with 4% paraformaldehyde and permeabilized in PBS containing 0.5% Triton X-100 and blocked in Block-Aid (Life Technologies) for 1 h at room temperature. Cells were then incubated with MF20 monoclonal antibody (DSHB) against MHC (1:40 dilution;) overnight at 4 degrees. Secondary antibody Alexa Fluor 488-conjugated secondary antibody (1:200 dilution; Life Technologies) for 1 h at room temperature. Cells were mounted with ProLong Gold antifade reagent with DAPI (4′,6-diamidino-2-phenylindole; Life Technologies).

### Immunoblotting

Cells were lysed in M-PER lysis buffer (Pierce Biotechnology). Lysates were denatured in 4× sample buffer at 70°C for 10 min, resolved on 4–12% NuPAGE gels (Life Technologies) and transferred onto PVDF (polyvinylidene fluoride) membranes. Membranes were blocked in 5% nonfat milk in TBST buffer (TBS with Tween-20) for 1 h at room temperature and probed with primary anti-HA antibody obtained from Covance (1:2,000 dilution). Bound antibodies were detected with peroxidase-labeled horse antibody to mouse IgG and visualized using enhanced chemiluminescence reagents (ThermoScientific).

### Expression analysis

Total cellular RNA was isolated using the RNAeasy mini kit (Qiagen). Cellular RNA (250ng) was in vitro transcribed, fragmented, hybridized and applied to Affymetrix Mouse 430A arrays according to the standard operating procedure of the Laboratory of Molecular Technology core facility (http://atp.ncifcrf.gov/genetics-and-genomics/laboratory-of-molecular-technology) and the manufacturer’s instructions (Affymetrix, Santa Clara, CA). For gene set enrichment analysis (GSEA), the normalized gene expression data were z-scored and ranked according to absolute fold-change expression over the control. GSEA analysis (http://www.broadinstitute.org/gsea/index.jsp) was performed using default parameter settings.

## Supporting Information

S1 Text Supporting text including supplemental methods. (DOCX)

S1 FigVerification of somatic calls and VAF using Ion Torrent targeted sequencing with 1997x coverage.(a) Verification accuracy: the accuracy is calculated as $1-|\hat{v}-v|/v$, where $\hat{v}$ is the VAF estimated by WGS and *v* is the VAF estimated by targeted deeper sequencing (1997x coverage). Green bar shows the average accuracy among the somatic mutations and whisker shows the variance. We perform the verification on two samples, and the average accuracy is 0.9. This high accuracy supports our inference of variant timing and subclonality. (b)-(c) are the scatter plots comparing the VAF estimated by WGS and the VAF verified by the targeted deep sequencing for each individual sample.(PDF)

S2 Fig Normal cell contamination correction corrects the observed unexpected allelic copy number status to expected status (allelic copy number is the product of LAF and total copy number). Expected status means integer allelic copy number while unexpected status means non-integer allelic copy number, which is a result of normal cell contamination or subclonal copy number changes. PFN rhabdomyosarcoma samples are marked by black fonts while PFP samples are marked by grey fonts. Blue bars show the number of chromosomes with unexpected status before normal cell contamination correction, while the red bars show how many of these chromosomes have expected status after the correction. Most chromosomes were corrected to expected status. A more detailed example is given in S3 Fig.
(PDF)

S3 Fig An example of normal cell contamination correction. For sample RMS2051, (a) and (c) show the VAF distribution of somatic mutations on chromosomes without aneuploidy, before and after normal cell contamination correction, respectively; (b) and (d) show the scatter plot of copy number and LAF of each chromosome, before and after normal cell contamination correction, respectively. Form (a), the observed VAF of somatic mutations (on chromosomes without aneuploidy) is distributed around 0.46, less than the expected value of 0.5 when there is no normal cell contamination. From (c), we found that the correction method has made the VAF distributed around the expected value of 0.5. Meanwhile, the normal cell contamination correction has changed the unexpected statuses of copy number and LAF of several chromosomes (represented by red dots) to expected statuses (represented by blue crosses). In this example, unexpected status means non-integer allelic copy number which happens due to normal cell contamination or subclonal copy number changes. For example, in (b), chromosome 3 and 16, the observed copy number = 2 and LAF = 0.04 indicates a non-integer copy number of the lesser allele. After normal cell contamination correction, the two chromosomes are with 2 copies and 0 LAF as shown in (d), indicating an integer copy number of the lesser allele.
(PDF)

S4 Fig Distribution of VAF of somatic mutations for 44 rhabdomyosarcoma samples. For samples without whole genome duplication (mostly PFN rhabdomyosarcoma), we plot VAF distribution on chromosomes with diploidy “AB”; for samples with whole genome duplication (mostly PFP-RMS), we plot VAF distribution on chromosomes with tetrasomy “AABB”. Each row shows 5 samples. Each row has two sub rows: the upper one shows the VAF distribution before normal cell contamination correction and the lower one shows the distribution after normal cell contamination. We can see that most samples have VAF centered on 0.5 in diploidy chromosomes, or VAF centered on 0.5 and 0.25 on tetrasomy chromosomes. Additionally, for some samples, we observed a small amount of mutations have VAF lower than expected. This finding indicates that these mutations are present only on a part of tumor cells (subclonal mutations).
(PDF)

S5 Fig Flow chart of the analysis method. The method is based on called copy number status and somatic mutations. First we used copy number and LAF to infer subclonal copy number changes. Then we use the distribution of VAF of somatic mutations to infer subclonal mutations. After that, timing of the aneuploidy is done by investigating the multi-modality VAF distribution. The occurrence time of aneuploidy is used to confine the occurrence time of somatic mutations. Finally, we summarize all the variant timing and subclonal changes to build the cancer evolutionary history.
(PDF)

S6 Fig Capability of our method in detecting mutations with small VAF. Somatic mutations in the 44 RMS samples were grouped according to their VAF (x axis). For each group, the number of mutations is denoted by green dots and total coverage at each mutation site is denoted by the red bars.
(PDF)

S7 Fig Illustration of analysis method. (a) Allele “A” gets duplicated at a certain time (indicated by the purple box). If a mutation occurred on allele “A” before the duplication (the pink circle), its mutant copy gets duplicated as well and thus the VAF is 2/3. On the contrary, if a mutation occurred after the duplication (the light green circle), there is only one mutant copy and the VAF is 1/3. The occurrence time of the duplication can be inferred by comparing the number of mutations with VAF = 1/3 to the number of mutations with VAF = 2/3. (b) illustrates another example where the chromosome has an “LOH+duplication” event.
(PDF)

S8 Fig Robustness of cancer evolutionary history estimation. Mutation accumulation speed is estimated when inferring the timing of genomic variants. Since our method estimates the mutation accumulation speed for individual chromosome groups (with unique copy number, LAF and somatic VAF distribution) independently, the consistent estimates of mutation accumulation speed from different chromosome groups can be an indicator of the robustness of our method. (a) shows that the mutations speed estimated independently from different chromosome groups of sample RMS2110 are consistent—standard deviation is 0.063, <1/10 of the mean 0.6791 (per megabase across the cancer lifespan). Such consistency is further shown in (b), by comparing the number of mutations accumulated on each chromosomes and the length of the chromosomes. As expected, the number of accumulated mutations is largely associated with the length of chromosomes (coefficient of determination r^2^ = 0.9985), indicating strong robustness of the estimation. (c) The robustness is observed on multiple other samples which also have multiple chromosome groups to infer the mutation speed independently. The well fitted regression lines and high coefficient of determination confirm the robustness of the method.
(PDF)

S9 Fig Summary of evolutionary history of 44 rhabdomyosarcoma. The evolutionary history timelines were built by inserting the timing of recurrent lesions, in percentage, into the cancer lifespan. The observed lesions are marked by different colors. (a) PFN samples. (b) PFP samples.
(PDF)

S10 Fig Loss of heterogeneity regions on chromosome 11 and 17. (a) 11p LOH regions for rhabdomyosarcoma samples sequenced by whole genome sequencing. (b) 11p LOH regions on a larger cohort (totally 117 samples by WGS and Illumina SNP array; the figure only show those samples with 11p LOH) indicate that *IGF2* is the minimal intersection. (c) chromosome 17 LOH regions for rhabdomyosarcoma samples sequenced by whole genome sequencing. (d) chromosome 17 LOH regions on the larger cohort overlap at two island regions centered around *TP53* and *NF1*, respectively. (e) chromosome 11 and 17 LOH is usually accompanied with chromosome duplication or copy gain.
(PDF)

S11 Fig 11p15.5 LOH (coupled with copy gain) is an early event that is most recurrent in PFN rhabdomyosarcoma. Green triangles represent the molecular time at biopsy, measured by the number of accumulated somatic mutations; purple squares represent the molecular time at 11p15.5 LOH event, measured by the estimated percentage of molecular time when the LOH event happened, multiplied by the molecular time at biopsy; orange bars represent the age of patients at biopsy.
(PDF)

S12 Fig Circos plots of typical RMS genomes. (a)-(c) are PFN samples and (d-f) are PFP samples. Gene symbols indicate genes with nonsynonymous mutations. Tracks from outermost to innermost: Somatically mutated genes, karyotype, copy number (dark blue bars), lesser allele fraction (green bar), loss of heterogeneity indicator (dark green dots), intensity of heterozygous mutations (orange bar) and homozygous mutations (blue bar), junctions or chromosomal rearrangement (grey lines for intra-chromosome rearrangement and orange lines for inter-chromosome rearrangement).
(PDF)

S13 Fig Whole genome duplication in PFP tumors. We use a PFP rhabdomyosarcoma sample NCI0040 as an example. (a) shows the VAF of somatic mutations on chromosomes with LAF = 0.5. The VAF of somatic mutations is distributed as a bi-modal normal mixture, centered on 0.25 and 0.5, respectively. Therefore, it is likely that the chromosomes are of tetrasomy—a mutation with 0.25 VAF is expected to have mutant on 1 out of 4 copies. It is also possible that the mutations of 0.25 VAF come from a subclone that is present in 50% of tumor cell; however such phenomenon occurs in 17 PFP tumors and it is unlikely that all these 17 tumors have the same subclonal composition. Interestingly, by timing the tetrasomy chromosome by chromosome, we found that the tetrasomy duplication happens around the same time for all the chromosomes, as shown in (b), suggesting an endoreduplication event.
(PDF)

S14 Fig Protein and position of the *PKN1* mutations. (PDF)

S1 Table Cancer evolutionary history of 44 RMS samples. Listed are fusion status, sample ID, age, normal cell contamination rate, occurrence time of whole genome duplication, number of clones, occurrence time of chromosomal aneuploidy, and occurrence time of nonsynonymous mutations. For aneuploidy, timing is listed followed by ploidy status, for example, "30% aab" means at 30% molecular time the chromosome ploidy changed to the trisomy "aab". For samples with whole genome duplication, only chromosomes with ploidy status different from tetrasomy "aabb" were listed. For mutations, each grid shows the time interval of occurrence.
(XLSX)

S2 Table Candidate list of somatic subclonal mutations. Annotations are listed for these mutations. Results of gene ontology analysis were also listed.
(XLSX)

S3 Table Timing of PAX fusion. Inferred occurrence timing of the PAX fusion event in PFN RMS samples is listed.
(XLSX)

S4 Table Timing and expression of somatic mutations. Somatic mutations of which occurrence time are inferable are listed.
(XLSX)

S5 Table Significantly enriched gene sets by GSEA. (XLSX)
