# Supplementary material for: Clonality and Evolutionary History of Rhabdomyosarcoma
Source: PLoS Genet. 2015 Mar 13;11(3):e1005075. doi: 10.1371/journal.pgen.1005075 (PMC4358975; doi:10.1371/journal.pgen.1005075)
Supplement: S1 Text — (DOCX) [file pgen.1005075.s001.docx]

Text S1: Clonality and Evolutionary History of Rhabdomyosarcoma

Li Chen, Jack F. Shern, Jun S. Wei, Marielle E. Yohe, Young K. Song, Laura Hurd, Hongling Liao, Daniel Catchpoole, Stephen X. Skapek, Frederic G. Barr, Douglas S. Hawkins, Javed Khan.

Supplementary Methods

To study the evolutionary history of rhabdomyosarcoma (RMS), we performed an integrative computational analysis, using various information sources including germline single-nucleotide variants (SNVs), somatic single-nucleotide variants (somatic mutations), somatic copy number alterations (CNA) and junctions (mapped breaking points), to estimate the normal cell contamination rate, intra-tumor heterogeneity (subclonality) and the occurrence time of somatic alterations. The integrative analysis method is built on the principles that were used in (2, 14) with customized modifications that are necessary for analyzing the data in this study ([1-4](#_ENREF_1)).

***Normal Cell Contamination Correction***

The first step in our method is to correct normal cell contamination. The fraction of normal cells in the sample (normal cell contamination rate) can be detected by comparing the variant allele fraction (VAF) of germline single-nucleotide variants to that of somatic mutations. When no copy number alteration is observed, the VAF of a heterozygous germline SNV is equal or larger than that of a somatic mutation, since germline SNVs exist in both tumor and normal cells while a somatic mutations exist only in tumor cells. This difference is utilized to detect the normal cell contamination rate (Supplementary Figures S2-S3). In detail, we first pick up somatic mutations on chromosomes without aneuploidy (with copy number equal to 2 and LAF equal to 0.5). Then we distinguish full-clonal mutations from subclonal mutations by selecting somatic mutations with higher VAF. After that we predict the existence of normal cell contamination based on whether VAF of full-clonal mutations distributes around 0.5 (there is no normal cell contamination) or a less value (there is normal cell contamination). The following model establishes the relationship between normal cell contamination rate $f_{n}$ and VAF of somatic mutations on chromosomes without aneuploidy,

$\left( 1-f_{n} \right)\cdot1=v_{s}\cdot2$ (1)

, where $v_{s}$ is the variant allele fraction of full-clonal (present in all tumor cells) somatic mutations. $v_{s}$ can be estimated from the observed variant allele fraction (VAF) distribution of somatic mutations, which is a mixture of full-clonal mutations and subclonal mutations (with smaller VAF) (see Supplementary Figure S4). This mixture can be modeled as a standard finite normal mixture that is later on dissected by expectation maximization algorithm ([5](#_ENREF_5)). The optimal number of clusters is decided by minimum description length ([6](#_ENREF_6)), an information criterion minimizing the data complexity (model complexity and goodness of data fitting), which usually corresponds to the best modeling of the data (Ockham's razor principle). $v_{s}$ is estimated as the center of the full-clonal VAF cluster and then $f_{n}$ is calculated from equation (1).

The estimated normal cell contamination rate $\hat{v_{s}}$ is then used to correct the observed values of allelic copy number and VAF of somatic mutations to the tumor-cell-only values. Our data illustrate that, due to normal cell contamination, many chromosomes have non-integer observed allelic copy number. After the normal cell contamination correction, these chromosomes become to have expected integer allelic copy number, illustrating the accuracy and efficacy of the correction (Supplementary Figure S2). A more illustrative example is given in Supplementary Figure S3, where chr9p and chr11 are observed as with 2 copies and 0.06 LAF; after the correction, chr9p and chr11 have 2 copies and 0 LAF, showing that these chromosomes are uniparental disomy.

***Inferring Occurrence Time of Genomic Alterations***

We applied the principles used in (2, 14) to infer the occurrence time of aneuploidy events. For a chromosome with somatic aneuploidy, the aneuploidy event will change the VAF of somatic mutations. The occurrence time of the aneuploidy can then be estimated by comparing the number of somatic mutations with different VAF values.

Supplementary Figure S7 illustrates the timing inference method. In Supplementary Figure S7 (a), a chromosome becomes triploidy “AAB” after a chromosomal duplication. The mutations accumulated on allele “A” before the duplication have two mutant copies (VAF=2/3) when sequenced, while mutations accumulated on “A” after the duplication and mutations accumulated on “B” throughout the whole cancer lifespan have only 1 mutant copy (VAF=1/3). Therefore, mutations can be classified into two groups based on VAF, and the numbers of mutations within these two groups reveal when the duplication happens.

Mathematically, on each copy of a chromosome, somatic mutation accumulation is modeled as a Poisson process ,

*N*~Poisson(*η t*) (2)

, where *N* is the number of accumulated somatic mutations, *η* is the expected accumulation speed of somatic mutation and *t* denotes the molecular time (in the unit of percentage during the tumor lifespan). Equation (2) is simply for a copy of a chromosome without aneuploidy. If a chromosome has aneuploidy, equation (2) will be adjusted according to ploidy. For the example in Supplementary Figure S7 (a)

*N*_1_~Poisson(*η t*+3 *η* (1-*t*)); *N*_2_~Poisson(*η t*) (3)

, where *N*_1_ and *N*_2_ are the numbers of somatic mutations with VAF=1/3 and 2/3, respectively, *η* is the speed of mutation accumulation, and *t* is percentage of molecular time before the duplication. Before the duplication there are only 1 copy for allele “A” and 1 copy for allele “B”, of each the total number of mutation is *ηt*; after the duplication, there are 3 copies and thus the number of mutations accumulated during this period is 3*η*(1-t)_._ Since mutations accumulated on allele “A” before *t* have VAF=2/3, the expectation of *N*_1_ is 3*η-*2*ηt* . Similarly, since mutations accumulated on allele “A” after *t* and those accumulated on allele “B” throughout whole tumor lifespan have VAF=1/3, the expectation of *N*_2_ is *η t*.

Based on *E*[*N*_1_]=3*η-*2*ηt* and *E*[*N*_2_]= *ηt*, a linear transform gives

$t=\frac{E[N_{2}]}{E\left[ N_{2} \right]+(E\left[ N_{1} \right]-E[N_{2}])/3}.$ (4)

Letting the observed number of mutations with 1/3 VAF and 2/3 VAF be *n*_1_ and *n*_2_, respectively, *t* and *η* are estimated as

$\hat{t}=\frac{n_{2}}{n_{2}+(n_{1}-n_{2})/3}$ ; $\hat{\eta}=\frac{{n_{1}+2n}_{2}}{3}$. (5)

Furthermore, the inferred occurrence time of aneuploidy can be used to confine the occurrence time of somatic mutations. For instance, if the duplication happens at *t*, mutations with 2/3 VAF should be accumulated before *t* (Supplementary Figure S7) and their occurrence time is confined within [0,$\hat{t}$].

***Intra-tumor Subclonality Dissection***

We employed both VAF of somatic mutations and allelic copy number of chromosomes to dissect subclonal tumor cell populations.

*a. Estimating Subclonality by Mutations*

The estimation of subclonality depends on the fact that a subclonal mutation tends to have lower VAF than full-clonal mutations. A typical scenario is on chromosomes without aneuploidy, where heterozygous full-clonal mutations have VAF = 0.5 while subclonal mutations have VAF<0.5. This difference was observed in some samples (see Figure 2b, samples RMS217 and RMS216) where there are clearly two VAF clusters, corresponding to full-clonal and subclonal mutations, respectively. Using the clustering method and model selection method mentioned in “*Normal Cell Contamination Correction*” section, we can identify subclonal mutations.

However, the VAF criterion is insufficient to detect subclones, due to the following reasons. First, somatic mutations with similar VAF may come from different subclones that include similar amount of tumor cells. In this case we may underestimate the number of subclones. Second, in addition to the amount of tumor cells possessing the mutation, VAF is also affected by the chromosome ploidy. A low VAF does not necessarily mean there is subclonality – it may be caused by aneuploidy as well. For example, a somatic mutation on a triploidy “AAB” chromosome with VAF=0.33 may be full-clonal with allele “B” mutated, or subclonal (present in 50% of tumor cells) with both copies of allele “A” mutated. In fact, most of the low-VAF mutations in our dataset are found in chromosomes with aneuploidy. Third, somatic mutations with smaller VAF need deeper sequencing to be detected. Under certain sequencing coverage, subclonal mutations in small subclones are not necessarily detectable. Therefore, the VAF-based estimation is a necessary but not sufficient criterion to infer subclonality.

*b. Estimating Subclonality by Copy Number Changes*

As we discussed in last section, due to the complex nature of genomic subclones and the limited sequencing coverage, only using VAF criterion is not sufficient to accurately detect subclones. Therefore we also use allelic copy number to detect subclones. For each chromosome, we investigated the joint status of copy number and LAF, as shown in Figure 1b. When all tumor cells have the same allelic copy number, the observed allelic copy number is an integer (corresponding to blue crosses in Figure 1b). If the observed allelic copy number fall out of the integer status, the tumor has subclones with different allelic copy number.

To infer the allelic copy number of subclones, we extrapolate the observed non-integer allelic copy number to integers. For example, a tumor with observed copy number = 2.5 is possibly a mixture of a subclone with copy number = 2 and another subclone with copy number = 3. This extrapolation has unlimited possibilities. A heuristic search strategy is designed to search on these possibilities following the Occam's razor principle – we begin the search from copy number and LAF values that are closest to our observation. For instance, if a chromosome has an observed copy number as 2.2 and an observed LAF as 0.45, we begins the search at a major subclone with 2 copy and 0.5 LAF, and then search for the minor subclone with >2.2 copy and <0.45 LAF, where the closest possibility is triploidy “AAB”. Mathematically, supposing that the major subclone is present in a fraction *η* of tumor cells, the copy number of major subclone is *C*_1_, LAF is *L*_1_, the copy number of minor subclone is *C*_2_, LAF is *L*_2_, and the observed copy number of the whole tumor cell population is *C*_o_ and LAF is *L*_o_, we have

*ηC*_1_+(1- *η*)*C*_2_*=C_o_* (6)

*ηC*_1_*L_1_*+(1- *η*)*C*_2_*L_2_=C_o_ L_o_* (7)

. Equations (6) and (7) give two estimates of *η*. By comparing these two estimates, we are able to assess the goodness of the extrapolation. If the estimates do not match, we search for the second closest status for copy number and LAF of minor clone (copy number = 3; LAF=0). If we go through all the possibilities for the minor subclone and could not find a satisfactory one, we come back to change the status for copy number and LAF for major clone (the closest status is copy number = 2; LAF=0), and then restart the search for minor clone. This process is repeated until we find the extrapolation that gives consistent estimates of *η* from equation (6) and (7).

*c. Estimating Subclonality by Integrating Mutations and Copy Number Changes*

We integrate the strategies in section a and b to give a more accurate detection of subclones. The VAF distribution is clustered in two steps: an unsupervised step and a supervised step.

In the unsupervised step, the observed VAF distribution is modeled as the mixture of several normal-distributed statistics each corresponding to VAF of mutations occurring in a specific fraction of tumor cells at a specific period of cancer evolutionary history. Expectation maximization algorithm ([5](#_ENREF_5)) is applied to estimate mean and variance of each statistic. An information criterion, minimum description length ([6](#_ENREF_6)) is applied to identify the optimal number of clusters. This “optimal” number leads to the best compression of the data, which usually corresponds to the best modeling of the data (Ockham's razor principle). Since VAF of mutations from multiple subclones may have the same value, the “optimal” number is actually the lower bond of the number of subclones.

In the supervised step, we compare the estimates of subclones from VAF distribution to the estimates of subclones from copy number analysis. A principle is applied to decide whether the estimates are consistent: the VAF cluster centers have to fit the possible VAF values given by the ploidy (for example, triploidy gives possible VAF values as 1/3, 2/3 and 1). If the estimates do not fit, the heuristic search for subclonal copy number analysis has to move forward for another possibility. If the estimates fit each other, the current subclone ploidy is confirmed. We then refine the analysis by a classification step – we use the possible VAF values given by the ploidy as the center of the observed VAF clusters, and reapply expectation maximization algorithm (one step, without updating cluster centers) to calculate the mutations’ membership in each VAF cluster.

When a tumor sample has multiple subclonal events (*e.g.* subclonal aneuploidy on multiple chromosomes), it is a non-trivial task to decide whether these events occurred in the same subclonal tumor cell population. There are criteria proposed to partially address this problem (2). A simple criterion is to see whether different subclonal events are present in the same fractions of tumor cells. If not, we consider that these events occurred in multiple subclones, *i.e.* there is at least an additional subclone that only possesses the subclonal events with a larger fraction of cells.

***Accuracy of Cancer Evolutionary History Study***

The cancer evolutionary history analysis is performed independently for chromosomes with different allelic ploidy. As a part of the results, the speed of somatic mutation accumulation is estimated independently for these chromosomes. Therefore the robustness of the analysis method can be tested by whether such accumulation speed estimates are consistent, for different chromosomes within the same sample. By our analysis of the RMS cohort, we found that the estimates of the speed of somatic mutation accumulation are consistent across different chromosome groups. Supplementary Figure S8a and S8b show the result for sample RMS2110, where the number of somatic mutations for each chromosomes is mostly related to the length of the chromosome, with the coefficient of determination *r*^2^=0.9985. This high coefficient of determination illustrates the robustness of the analysis method, where there is little perturbation observed. The estimated mutation speed is 0.679±0.063 (mean ± standard deviation) per million bases across the cancer lifetime. We also tested the robustness for other samples, and observed a similar result. The result is shown in Supplementary Figure S8c.

Due to the limit of coverage depth, subclonal mutations with very small VAF may not be called. In order to know how small the VAF can be for a called mutation, we compared the VAF of called mutations in all the 44 samples and the total number of high-quality reads on the mutation sites. Supplementary Figure S6 shows that somatic mutations with smaller VAF need more high-quality reads to be called (red bars at left are higher). Somatic mutations with smaller VAF are usually more than those with larger VAF (green dots at left are generally higher, except for the leftmost one, with VAF<0.1). This finding coincides with the fact that heterozygous mutations and subclonal mutations (with smaller VAF) are more likely to happen than full-clonal homozygous mutations or duplicated mutations (with larger VAF). An exception is the mutations with VAF<0.1, which are far less than those called with 0.1<VAF<0.2. There are two possible explanations of the exception: (1) the subclones came out late (close to biopsy) so that many subclonal somatic mutations were accumulated in the limited time; (2) some mutations with VAF<0.1 are missed by the mutation caller due to the limit of coverage; in other words, more high-quality reads are in need to capture mutations with lower VAF.

**Supplementary Discussion**

***Subclonal somatic mutations***

Subclonal somatic events included mutations in COSMIC genes including *ABL1*, *BUB1B*, *CDK12*, *ERBB2*, *IGF2*, *KDR* and *SMARCA4*. Gene ontology for all these genes showed enrichment for those involved in cell adhesion (GO:0007155 p=0.02), DNA repair (GO:0006281 p=0.05) and cell migration (GO:0030335 p=0.07). Across the population, several aneuploidy events were recurrently found in the subclonal population, including gain of chr5, chr8, chr11, chr13, chr14, chr18 and chr19 (Supplementary Table S1).

**References**

2. Nik-Zainal S*, et al.* (2012) The life history of 21 breast cancers. *Cell* 149(5):994-1007.

14. Durinck S*, et al.* (2011) Temporal Dissection of Tumorigenesis in Primary Cancers. *Cancer Discovery* 1(2):137-143.

**Supplementary References**

1. Carter SL*, et al.* (2012) Absolute quantification of somatic DNA alterations in human cancer. *Nature biotechnology* 30(5):413-421.

2. Roth A*, et al.* (2014) PyClone: statistical inference of clonal population structure in cancer. *Nature Methods* 11(4):396-398.

3. Greenman CD*, et al.* (2012) Estimation of rearrangement phylogeny for cancer genomes. *Genome research* 22(2):346-361.

4. Chen L*, et al.* (2011) Tissue-specific compartmental analysis for dynamic contrast-enhanced MR imaging of complex tumors. *IEEE transactions on medical imaging* 30(12):2044-2058.

5. Hastie T, Tibshirani R, & Friedman J (2009) *The Elements of Statistical Learning: Data Mining, Inference, and Prediction.* (Springer).

6. Rissanen J (1978) Modeling by shortest data description. *Automatica* 14(5):465–658.
