# Supplementary figures and images for: Clonality and Evolutionary History of Rhabdomyosarcoma
Source: PLoS Genet. 2015 Mar 13;11(3):e1005075. doi: 10.1371/journal.pgen.1005075 (PMC4358975; doi:10.1371/journal.pgen.1005075)

**a**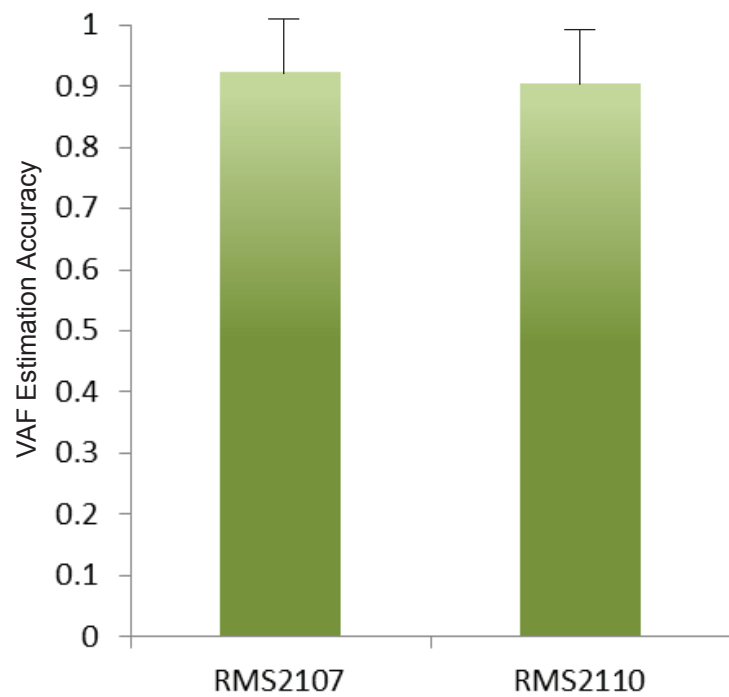**b**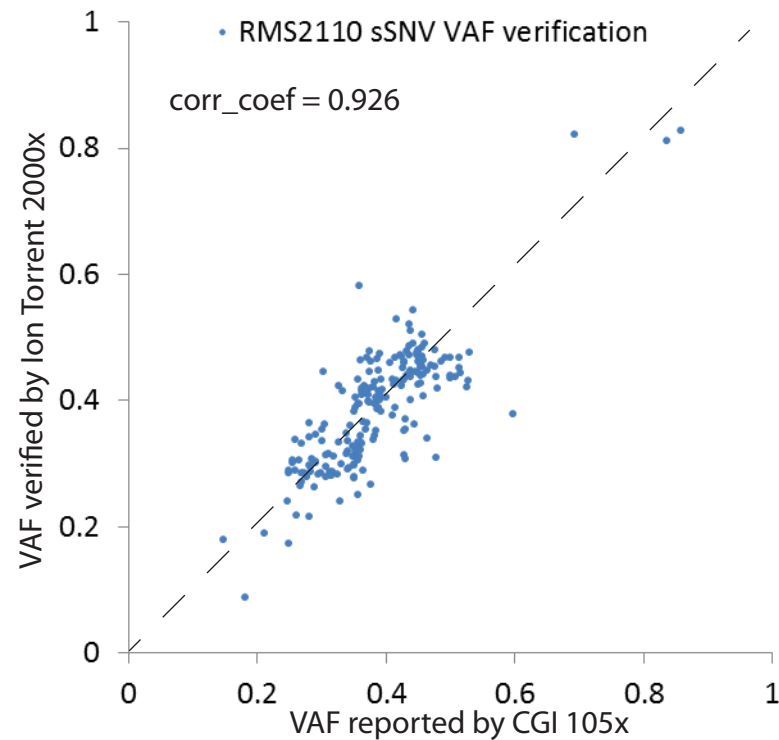**c**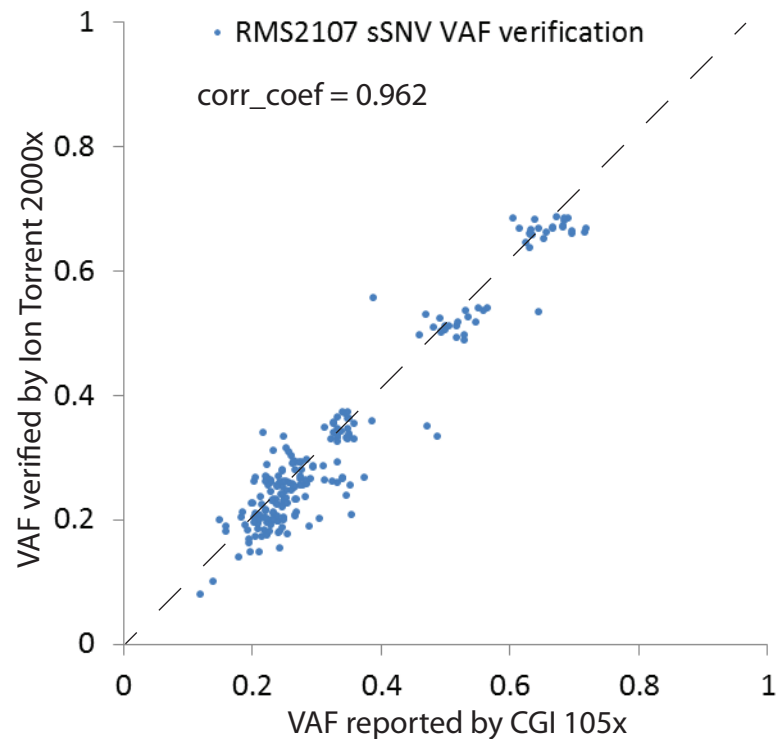

Supplement: S1 Fig — (a) Verification accuracy: the accuracy is calculated as 1-|v^-v|/v, where v^ is the VAF estimated by WGS and v is the VAF estimated by targeted deeper sequencing (1997x coverage). Green bar shows the average accuracy among the somatic mutations and whisker shows the variance. We perform the verification on two samples, and the average accuracy is 0.9. This high accuracy supports our inference of variant timing and subclonality. (b)-(c) are the scatter plots comparing the VAF estimated by WGS and the VAF verified by the targeted deep sequencing for each individual sample. (PDF) [file pgen.1005075.s002.pdf]

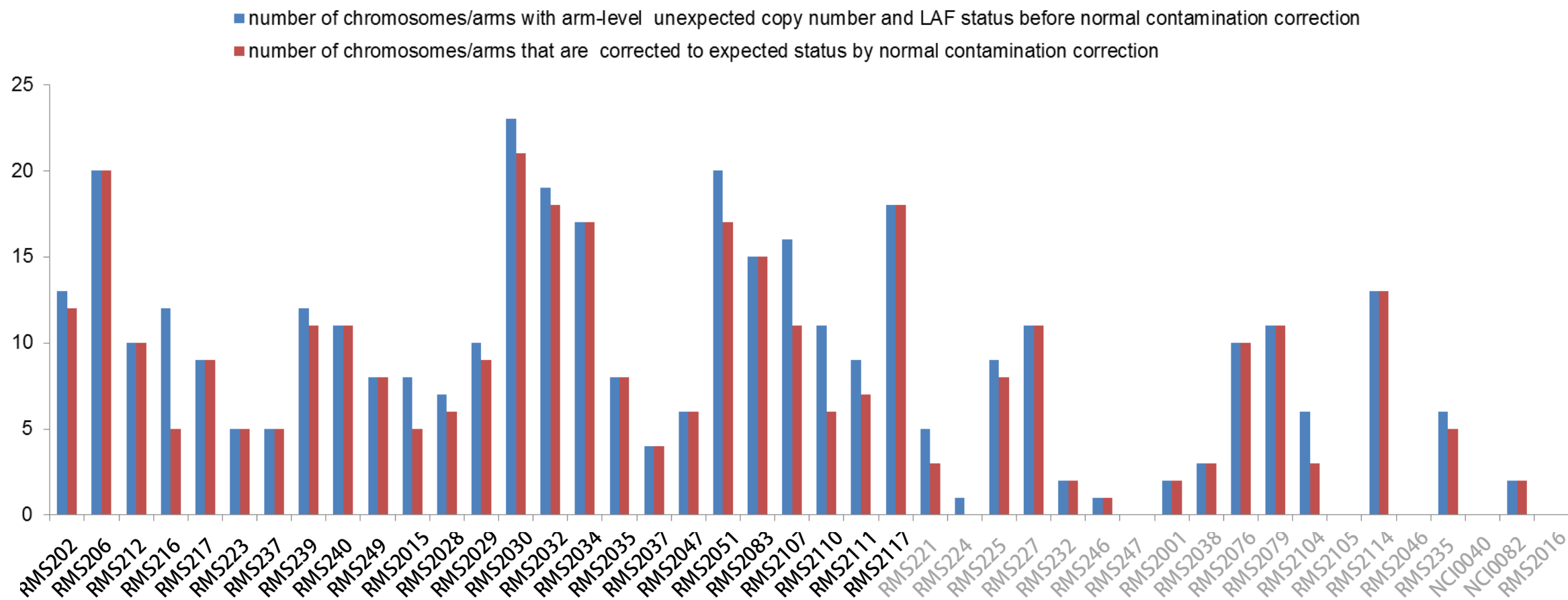

Supplementary Figure S2. Chen *et al.*

Supplement: S2 Fig — Expected status means integer allelic copy number while unexpected status means non-integer allelic copy number, which is a result of normal cell contamination or subclonal copy number changes. PFN rhabdomyosarcoma samples are marked by black fonts while PFP samples are marked by grey fonts. Blue bars show the number of chromosomes with unexpected status before normal cell contamination correction, while the red bars show how many of these chromosomes have expected status after the correction. Most chromosomes were corrected to expected status. A more detailed example is given in S3 Fig. (PDF) [file pgen.1005075.s003.pdf]

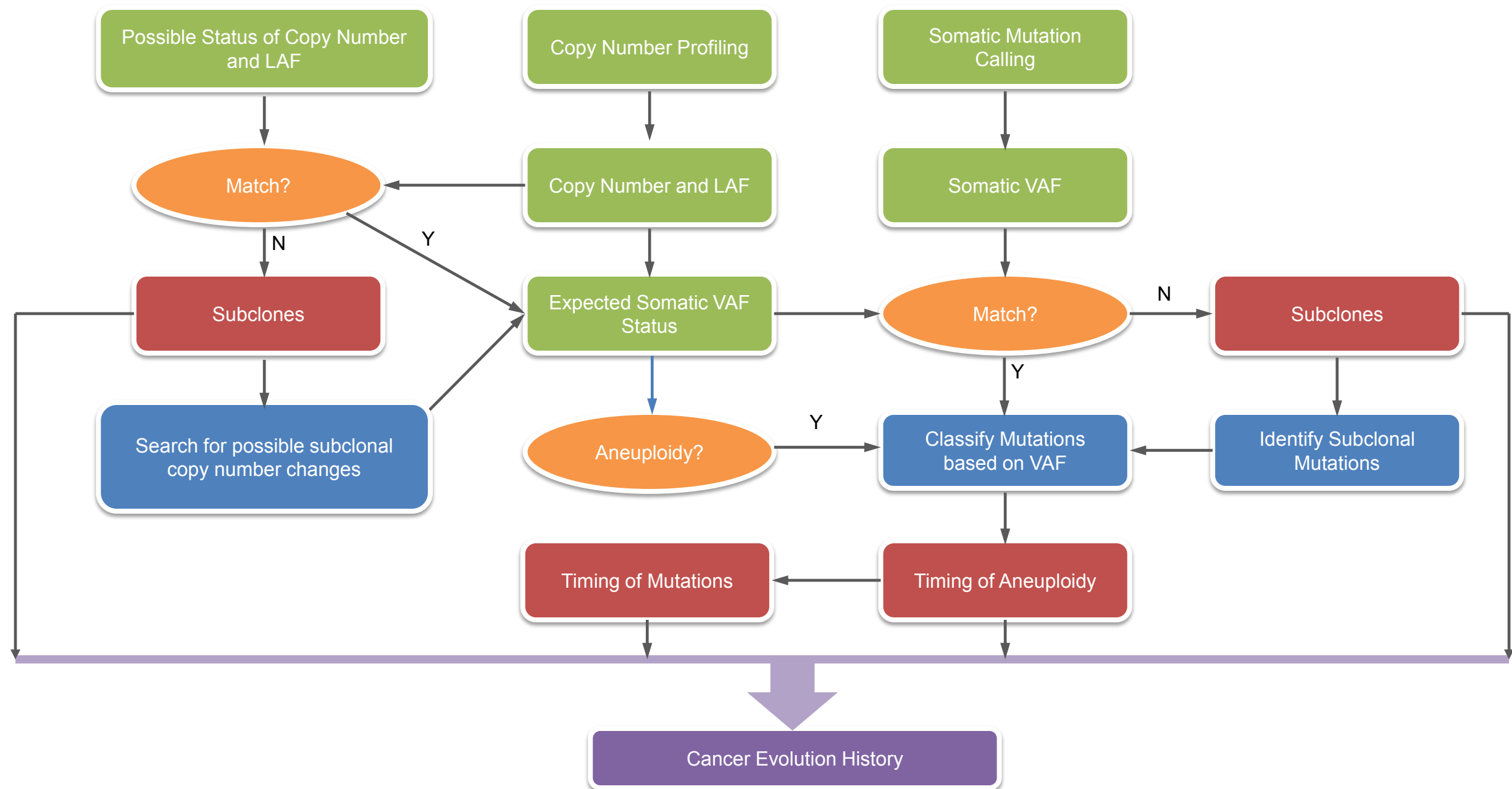

Supplementary Figure S5. Chen *et al.*

Supplement: S5 Fig — The method is based on called copy number status and somatic mutations. First we used copy number and LAF to infer subclonal copy number changes. Then we use the distribution of VAF of somatic mutations to infer subclonal mutations. After that, timing of the aneuploidy is done by investigating the multi-modality VAF distribution. The occurrence time of aneuploidy is used to confine the occurrence time of somatic mutations. Finally, we summarize all the variant timing and subclonal changes to build the cancer evolutionary history. (PDF) [file pgen.1005075.s006.pdf]

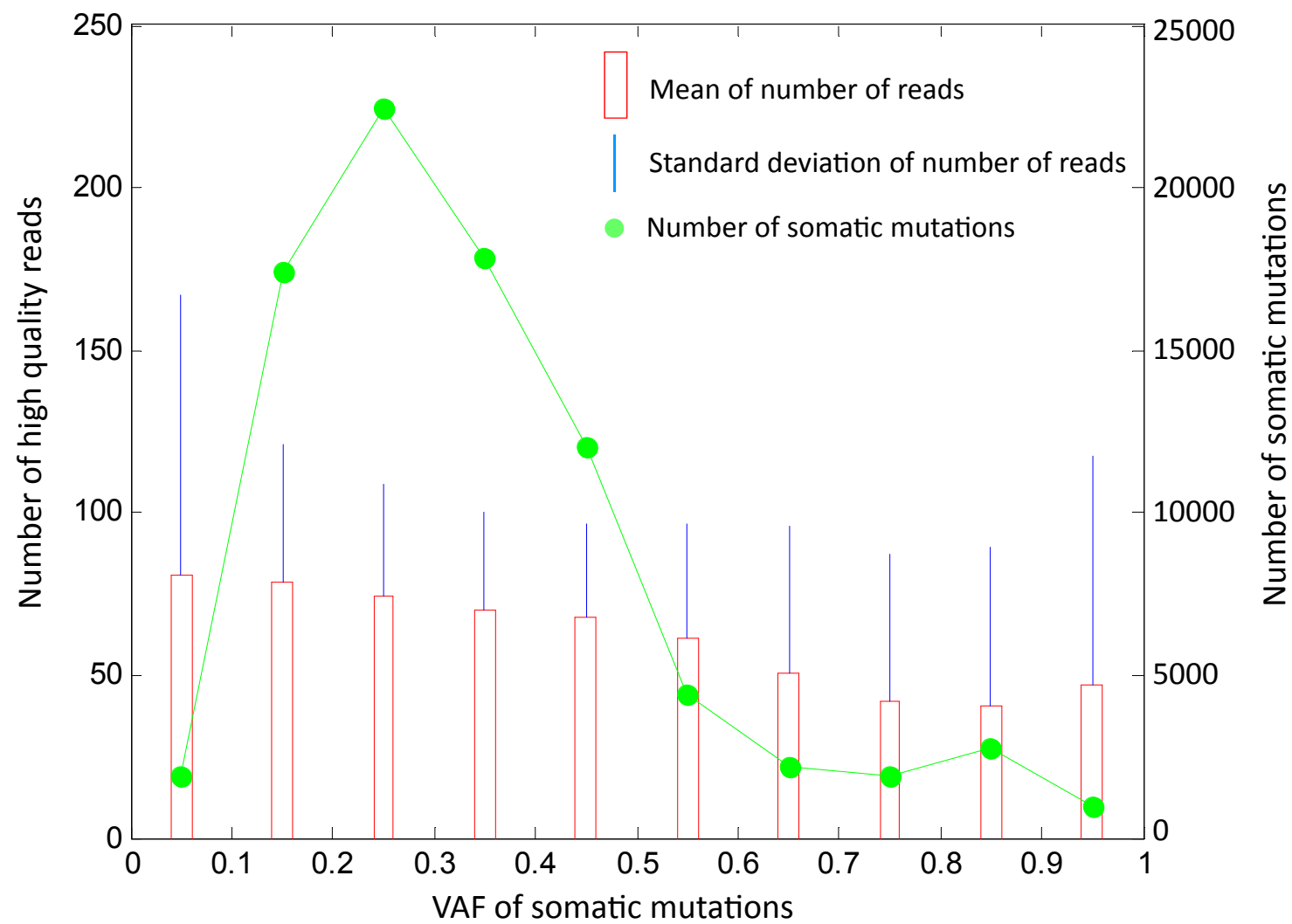

Supplementary Figure S6. Chen *et al.*

Supplement: S6 Fig — Somatic mutations in the 44 RMS samples were grouped according to their VAF (x axis). For each group, the number of mutations is denoted by green dots and total coverage at each mutation site is denoted by the red bars. (PDF) [file pgen.1005075.s007.pdf]

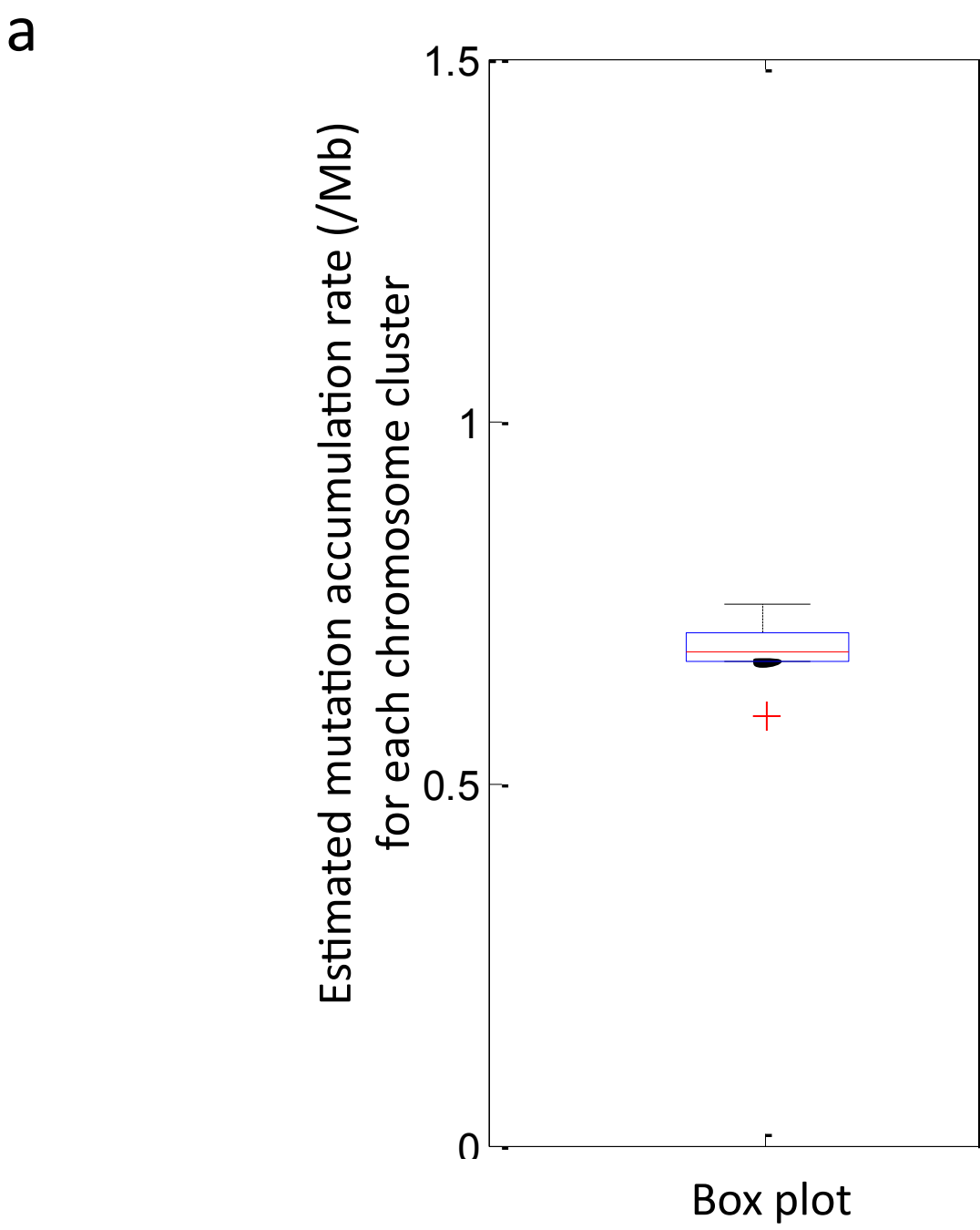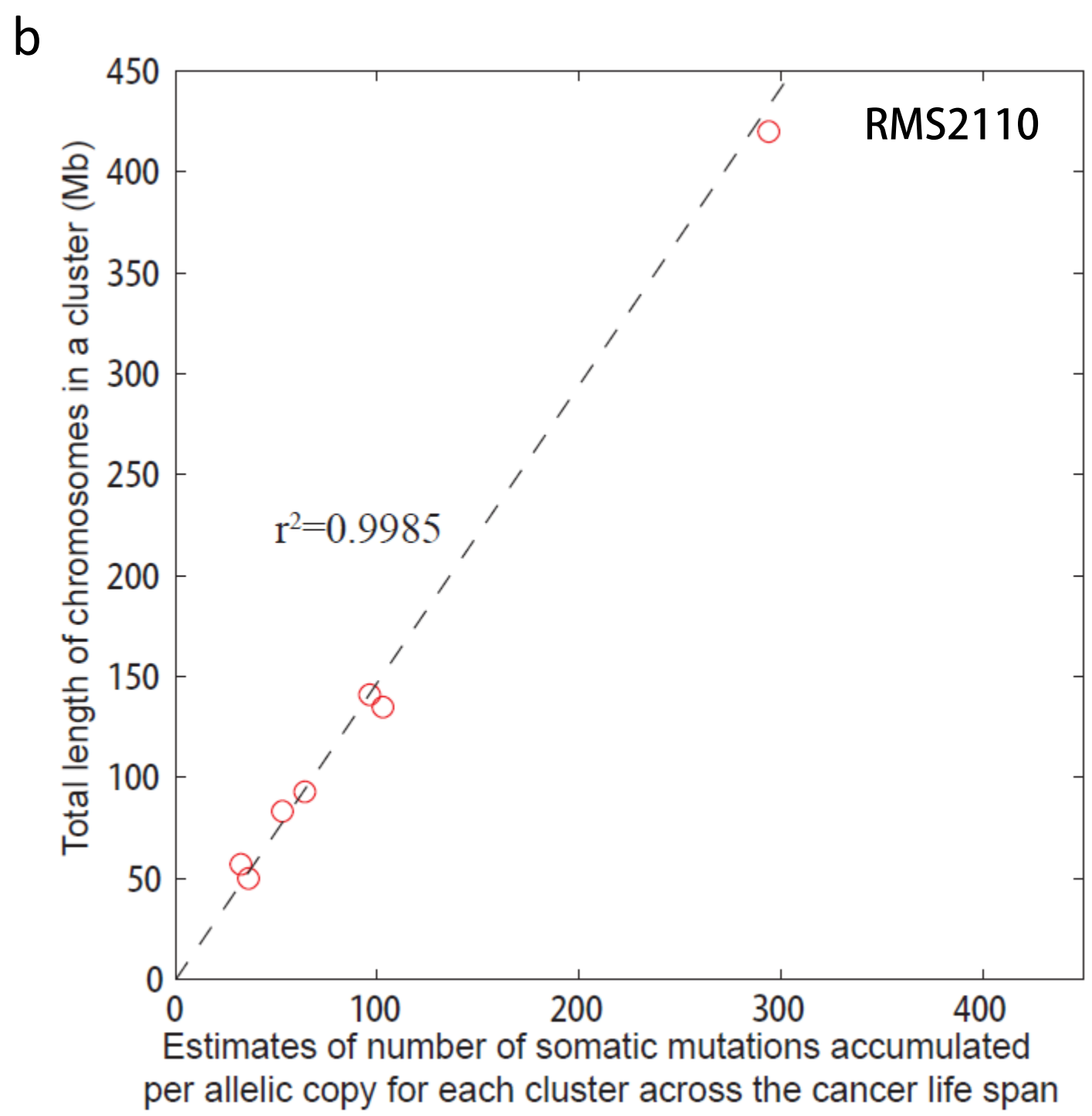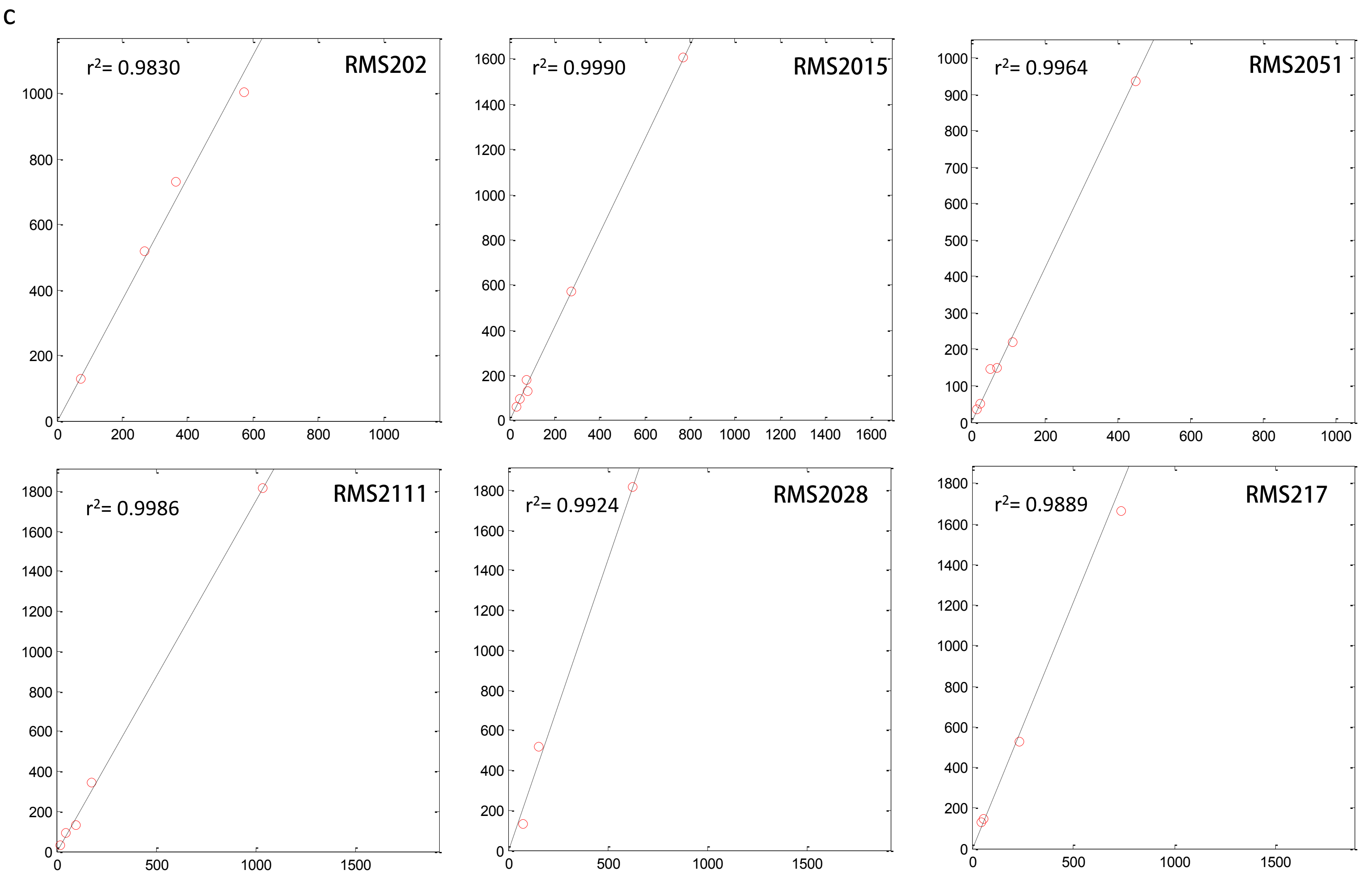

Supplementary Figure S8. Chen *et al.*

Supplement: S8 Fig — Mutation accumulation speed is estimated when inferring the timing of genomic variants. Since our method estimates the mutation accumulation speed for individual chromosome groups (with unique copy number, LAF and somatic VAF distribution) independently, the consistent estimates of mutation accumulation speed from different chromosome groups can be an indicator of the robustness of our method. (a) shows that the mutations speed estimated independently from different chromosome groups of sample RMS2110 are consistent—standard deviation is 0.063, <1/10 of the mean 0.6791 (per megabase across the cancer lifespan). Such consistency is further shown in (b), by comparing the number of mutations accumulated on each chromosomes and the length of the chromosomes. As expected, the number of accumulated mutations is largely associated with the length of chromosomes (coefficient of determination r2 = 0.9985), indicating strong robustness of the estimation. (c) The robustness is observed on multiple other samples which also have multiple chromosome groups to infer the mutation speed independently. The well fitted regression lines and high coefficient of determination confirm the robustness of the method. (PDF) [file pgen.1005075.s009.pdf]

a

## PAX-fusion-negative RMS

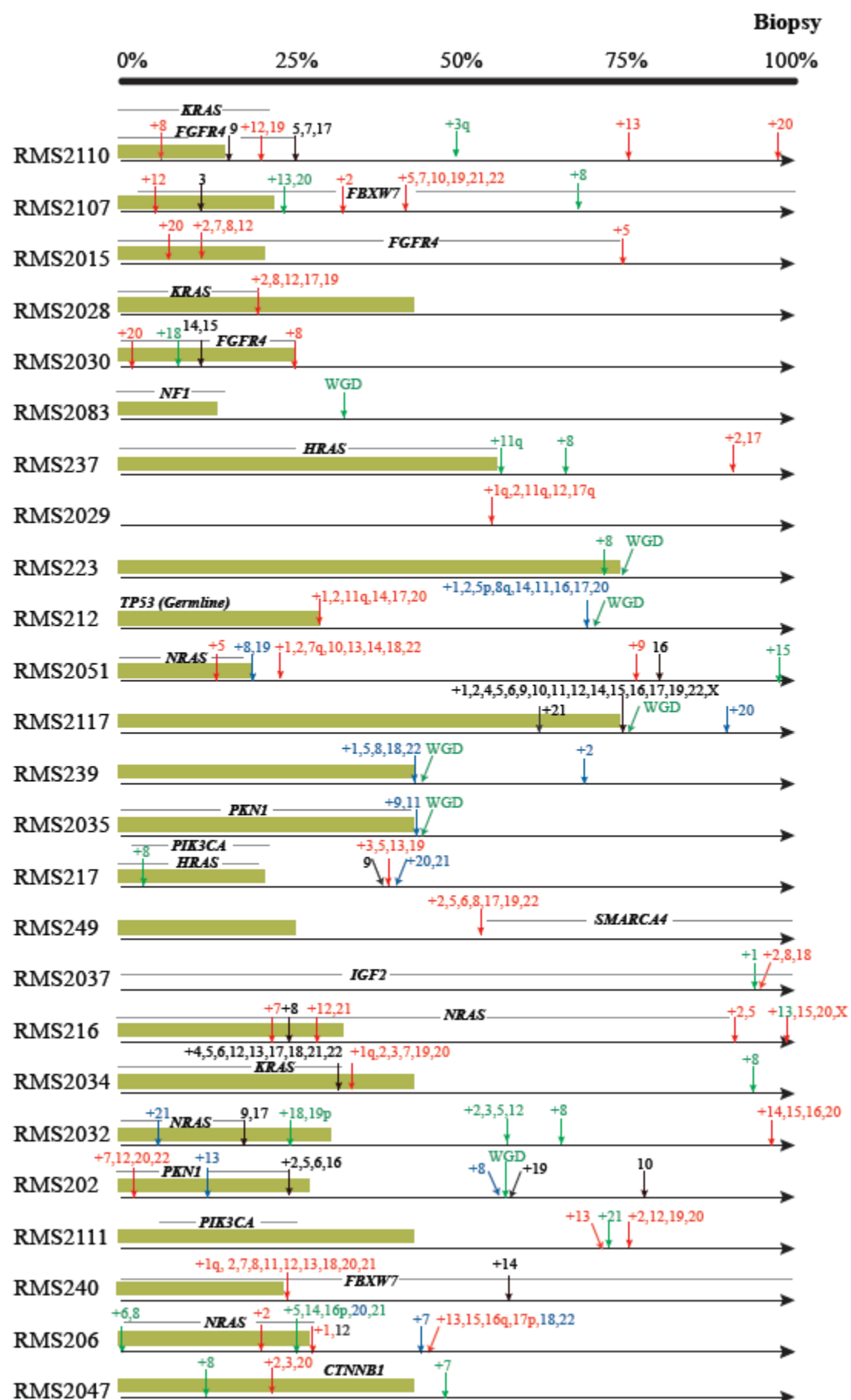

b

## PAX-fusion-positive RMS

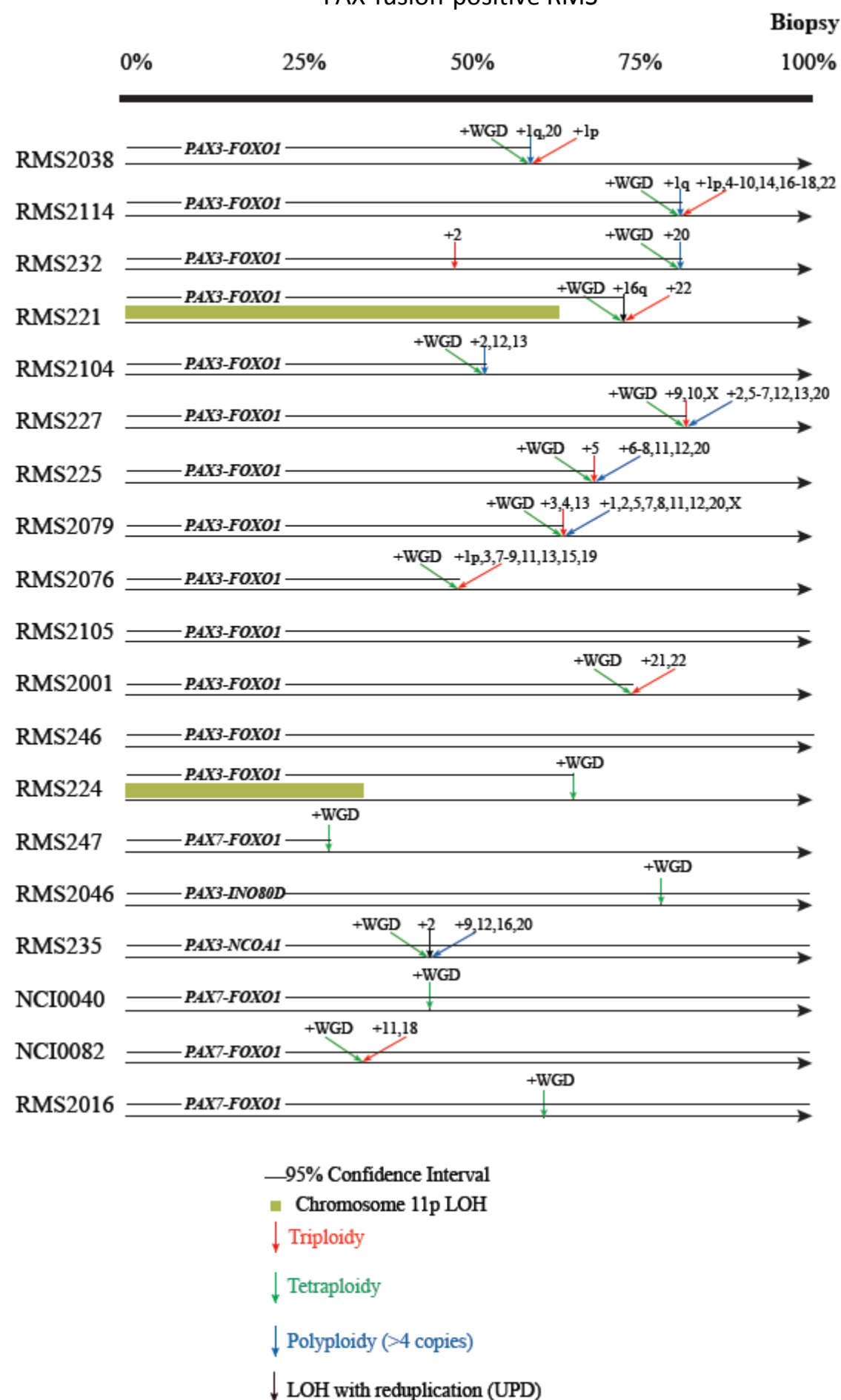

Supplement: S9 Fig — The evolutionary history timelines were built by inserting the timing of recurrent lesions, in percentage, into the cancer lifespan. The observed lesions are marked by different colors. (a) PFN samples. (b) PFP samples. (PDF) [file pgen.1005075.s010.pdf]

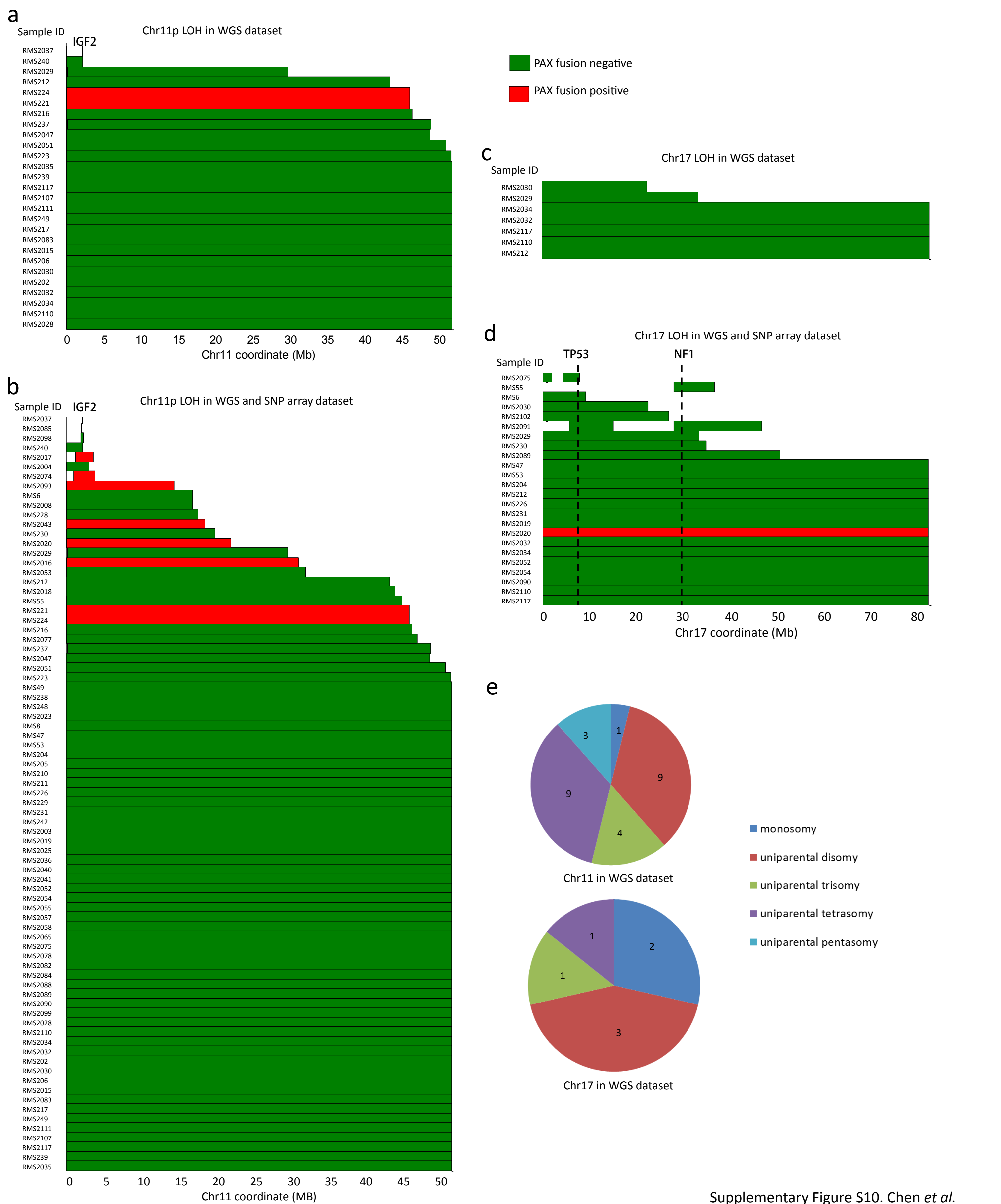

Supplementary Figure S10. Chen *et al.*

Supplement: S10 Fig — (a) 11p LOH regions for rhabdomyosarcoma samples sequenced by whole genome sequencing. (b) 11p LOH regions on a larger cohort (totally 117 samples by WGS and Illumina SNP array; the figure only show those samples with 11p LOH) indicate that IGF2 is the minimal intersection. (c) chromosome 17 LOH regions for rhabdomyosarcoma samples sequenced by whole genome sequencing. (d) chromosome 17 LOH regions on the larger cohort overlap at two island regions centered around TP53 and NF1, respectively. (e) chromosome 11 and 17 LOH is usually accompanied with chromosome duplication or copy gain. (PDF) [file pgen.1005075.s011.pdf]

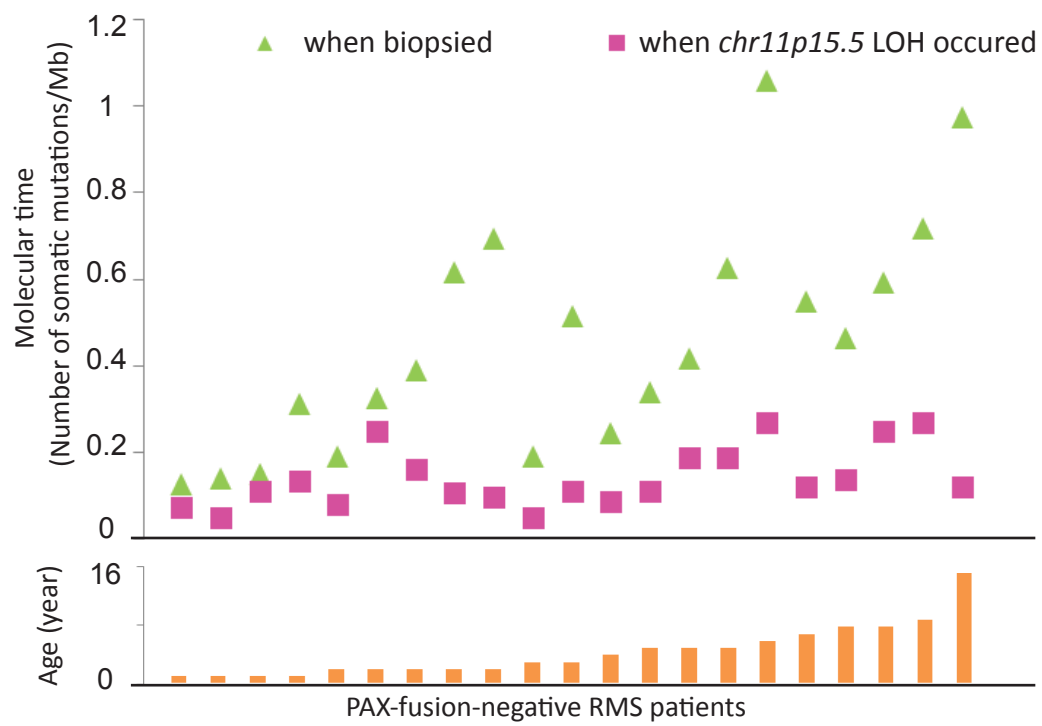

Supplementary Figure S11. Chen *et al.*

Supplement: S11 Fig — Green triangles represent the molecular time at biopsy, measured by the number of accumulated somatic mutations; purple squares represent the molecular time at 11p15.5 LOH event, measured by the estimated percentage of molecular time when the LOH event happened, multiplied by the molecular time at biopsy; orange bars represent the age of patients at biopsy. (PDF) [file pgen.1005075.s012.pdf]

(a)

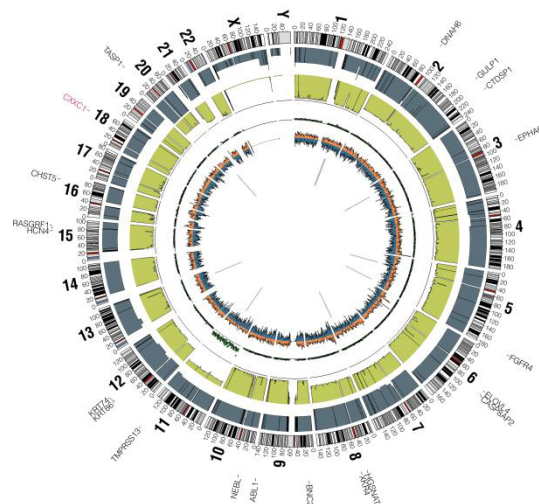

(b)

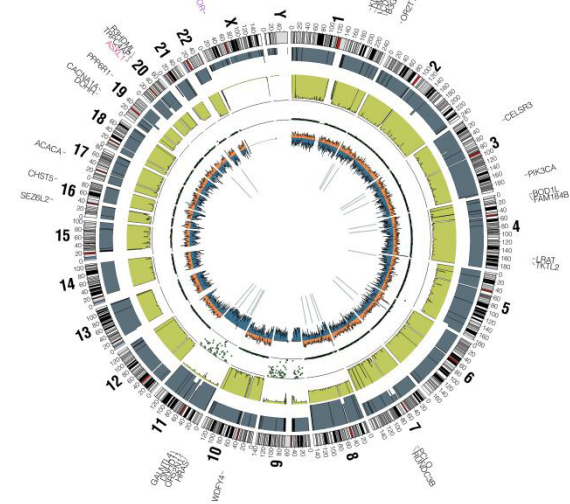

(c)

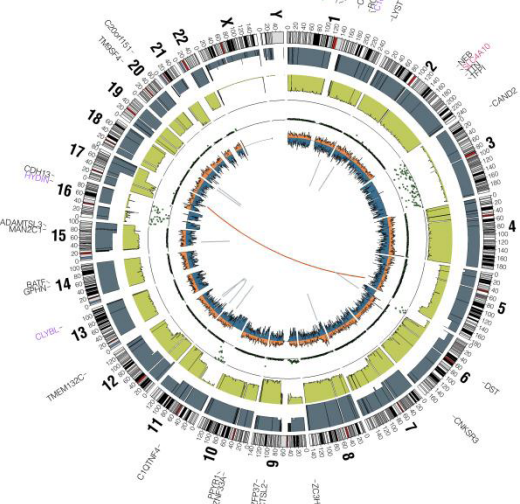

(d)

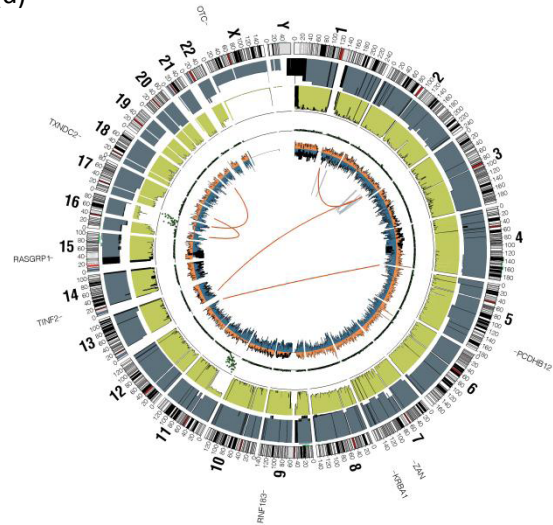

(e)

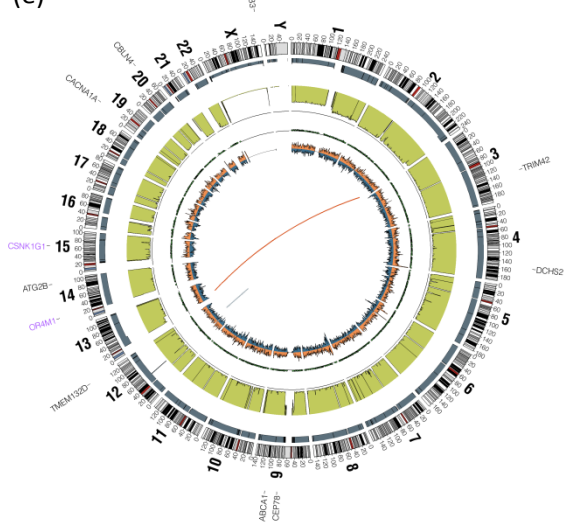

(f)

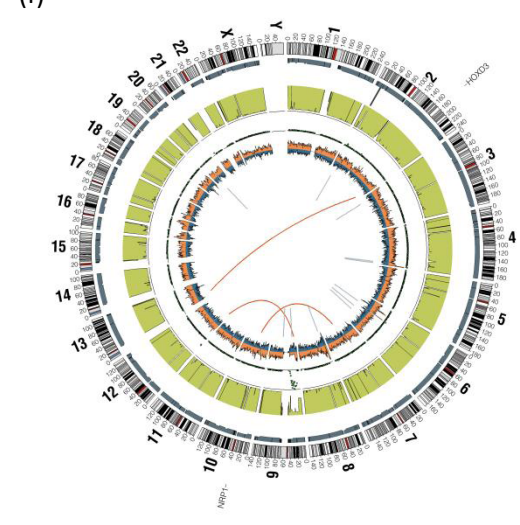Supplementary Figure S12. Chen *et al.*

Supplement: S12 Fig — (a)-(c) are PFN samples and (d-f) are PFP samples. Gene symbols indicate genes with nonsynonymous mutations. Tracks from outermost to innermost: Somatically mutated genes, karyotype, copy number (dark blue bars), lesser allele fraction (green bar), loss of heterogeneity indicator (dark green dots), intensity of heterozygous mutations (orange bar) and homozygous mutations (blue bar), junctions or chromosomal rearrangement (grey lines for intra-chromosome rearrangement and orange lines for inter-chromosome rearrangement). (PDF) [file pgen.1005075.s013.pdf]

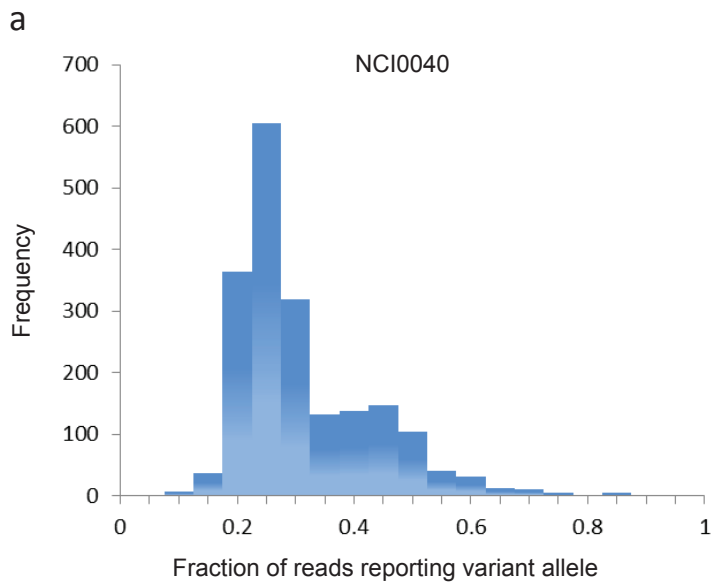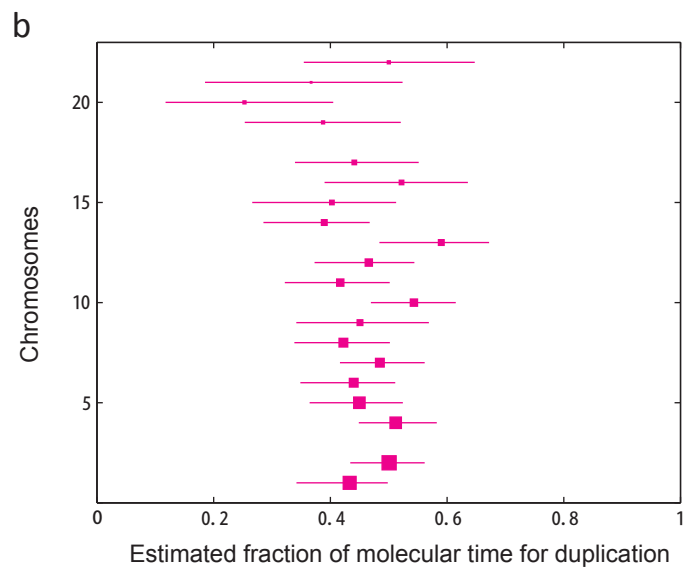

Supplementary Figure S13. Chen *et al.*

Supplement: S13 Fig — We use a PFP rhabdomyosarcoma sample NCI0040 as an example. (a) shows the VAF of somatic mutations on chromosomes with LAF = 0.5. The VAF of somatic mutations is distributed as a bi-modal normal mixture, centered on 0.25 and 0.5, respectively. Therefore, it is likely that the chromosomes are of tetrasomy—a mutation with 0.25 VAF is expected to have mutant on 1 out of 4 copies. It is also possible that the mutations of 0.25 VAF come from a subclone that is present in 50% of tumor cell; however such phenomenon occurs in 17 PFP tumors and it is unlikely that all these 17 tumors have the same subclonal composition. Interestingly, by timing the tetrasomy chromosome by chromosome, we found that the tetrasomy duplication happens around the same time for all the chromosomes, as shown in (b), suggesting an endoreduplication event. (PDF) [file pgen.1005075.s014.pdf]
