# Supplementary material for: Clonality and Evolutionary History of Rhabdomyosarcoma
Source: PLoS Genet. 2015 Mar 13;11(3):e1005075. doi: 10.1371/journal.pgen.1005075 (PMC4358975; doi:10.1371/journal.pgen.1005075)
Supplement: S3 Fig — For sample RMS2051, (a) and (c) show the VAF distribution of somatic mutations on chromosomes without aneuploidy, before and after normal cell contamination correction, respectively; (b) and (d) show the scatter plot of copy number and LAF of each chromosome, before and after normal cell contamination correction, respectively. Form (a), the observed VAF of somatic mutations (on chromosomes without aneuploidy) is distributed around 0.46, less than the expected value of 0.5 when there is no normal cell contamination. From (c), we found that the correction method has made the VAF distributed around the expected value of 0.5. Meanwhile, the normal cell contamination correction has changed the unexpected statuses of copy number and LAF of several chromosomes (represented by red dots) to expected statuses (represented by blue crosses). In this example, unexpected status means non-integer allelic copy number which happens due to normal cell contamination or subclonal copy number changes. For example, in (b), chromosome 3 and 16, the observed copy number = 2 and LAF = 0.04 indicates a non-integer copy number of the lesser allele. After normal cell contamination correction, the two chromosomes are with 2 copies and 0 LAF as shown in (d), indicating an integer copy number of the lesser allele. (PDF) [file pgen.1005075.s004.pdf]

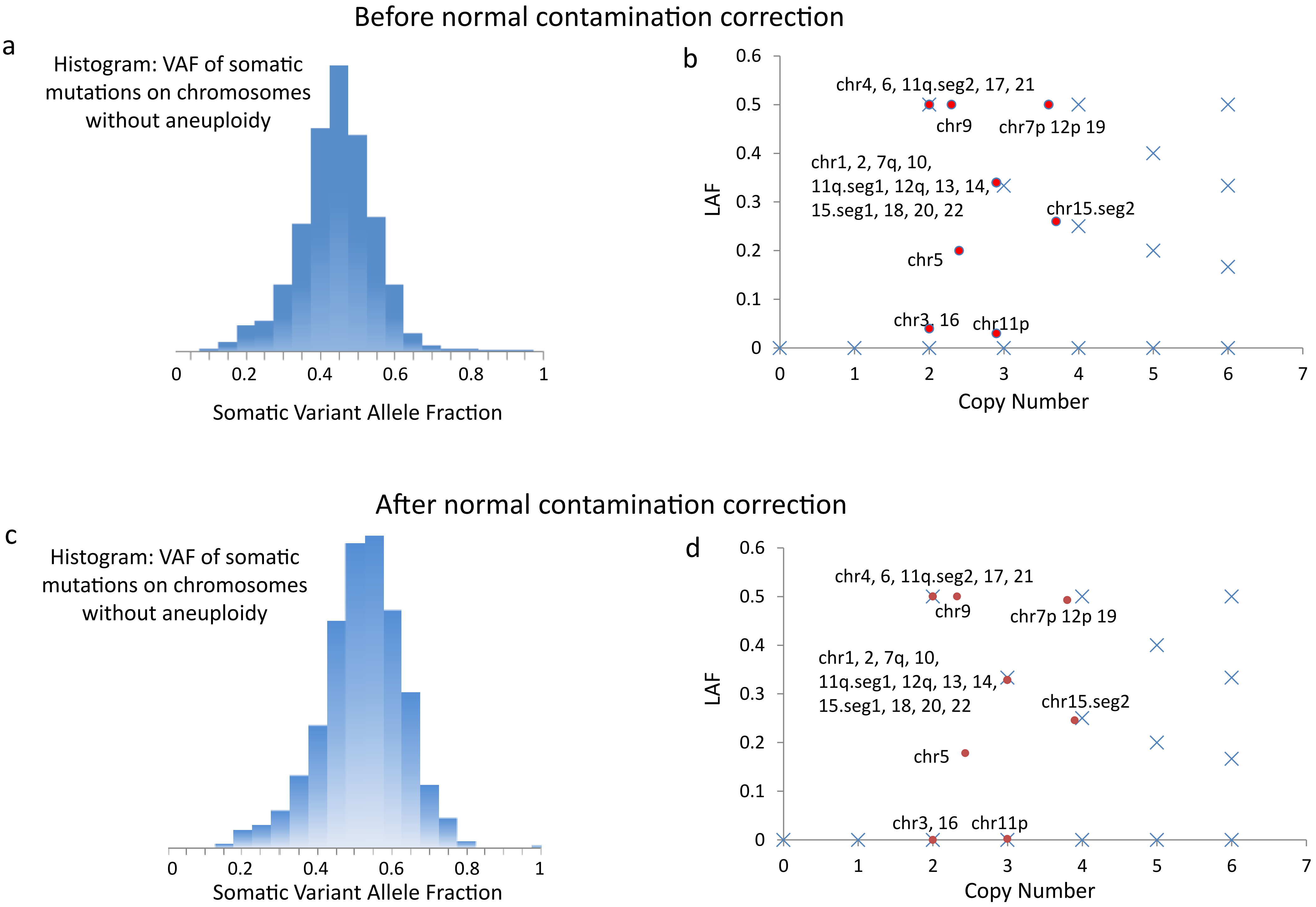

Supplementary Figure S3. Chen *et al.*
