# Supplementary material for: Clonality and Evolutionary History of Rhabdomyosarcoma
Source: PLoS Genet. 2015 Mar 13;11(3):e1005075. doi: 10.1371/journal.pgen.1005075 (PMC4358975; doi:10.1371/journal.pgen.1005075)

Somatic VAF distribution for Fusion Negative RMS: before and after NCR (normal contamination correction)
Each row has two subrows: the upper one is before NCR the lower one is after NCR

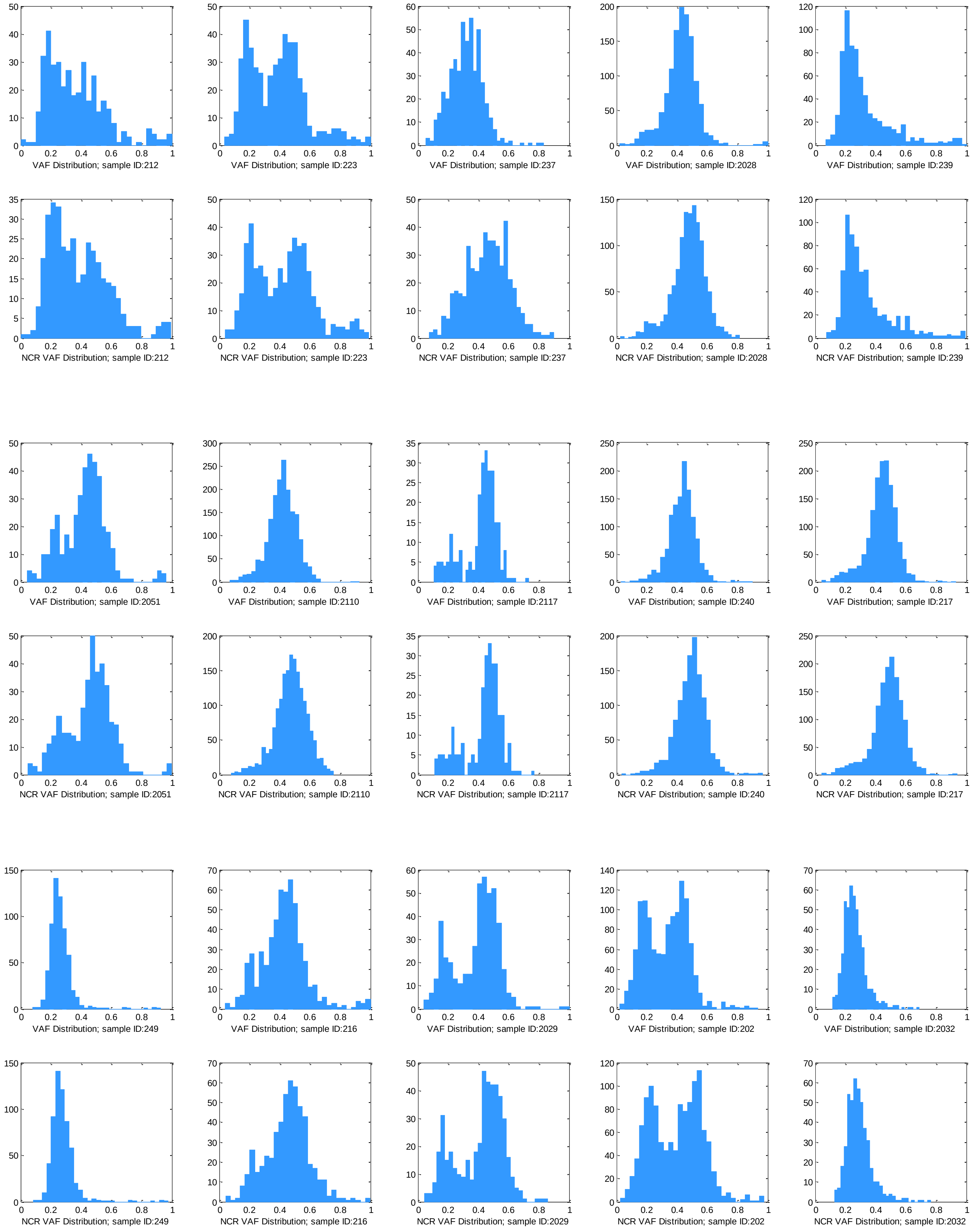

Somatic VAF distribution for Fusion Negative RMS: before and after NCR (normal contamination correction)

Each row has two subrows: the upper one is before NCR the lower one is after NCR

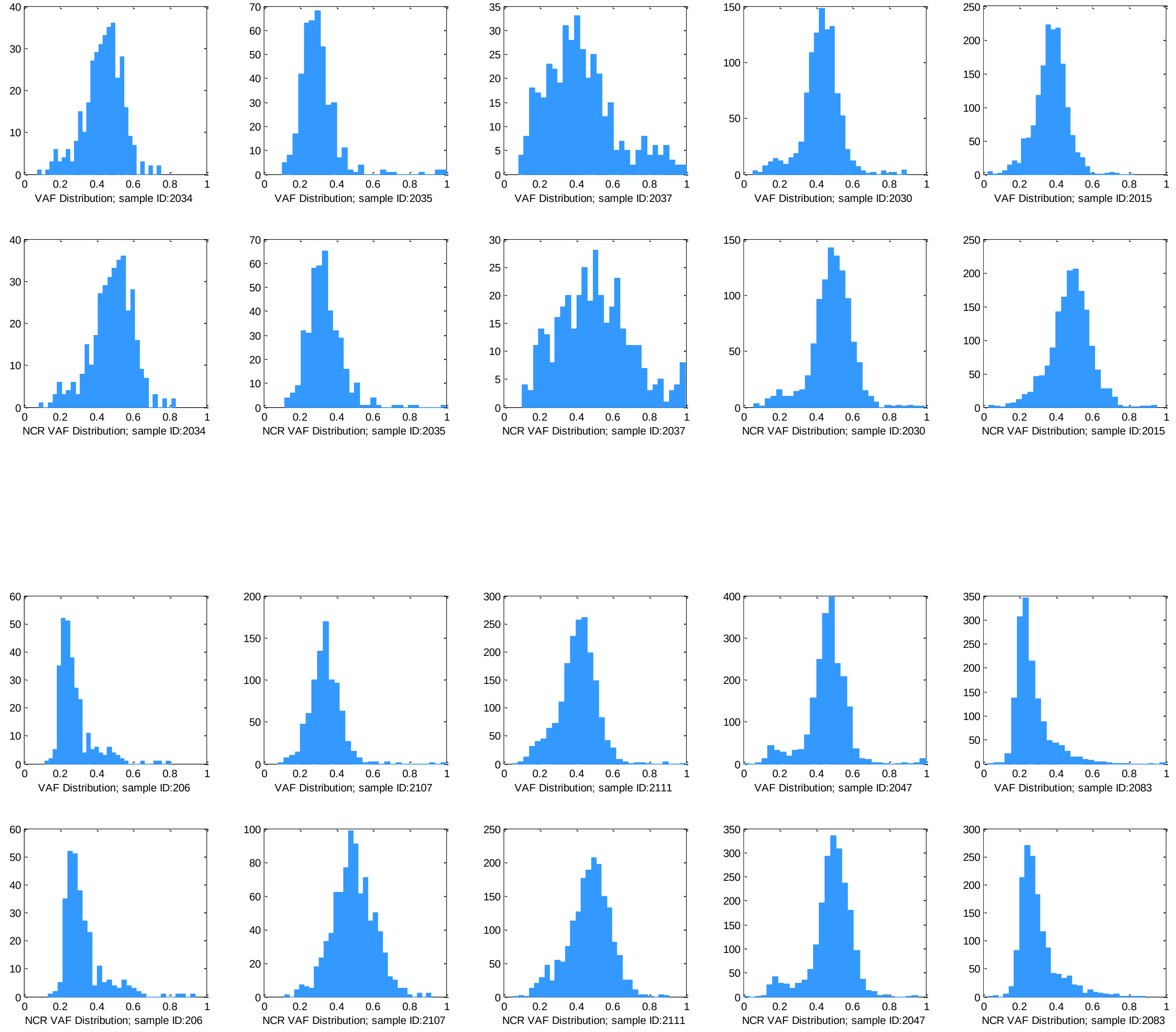

Somatic VAF distribution for Fusion Positive RMS: before and after NCR (normal contamination correction)
Each row has two subrows: the upper one is before NCR the lower one is after NCR

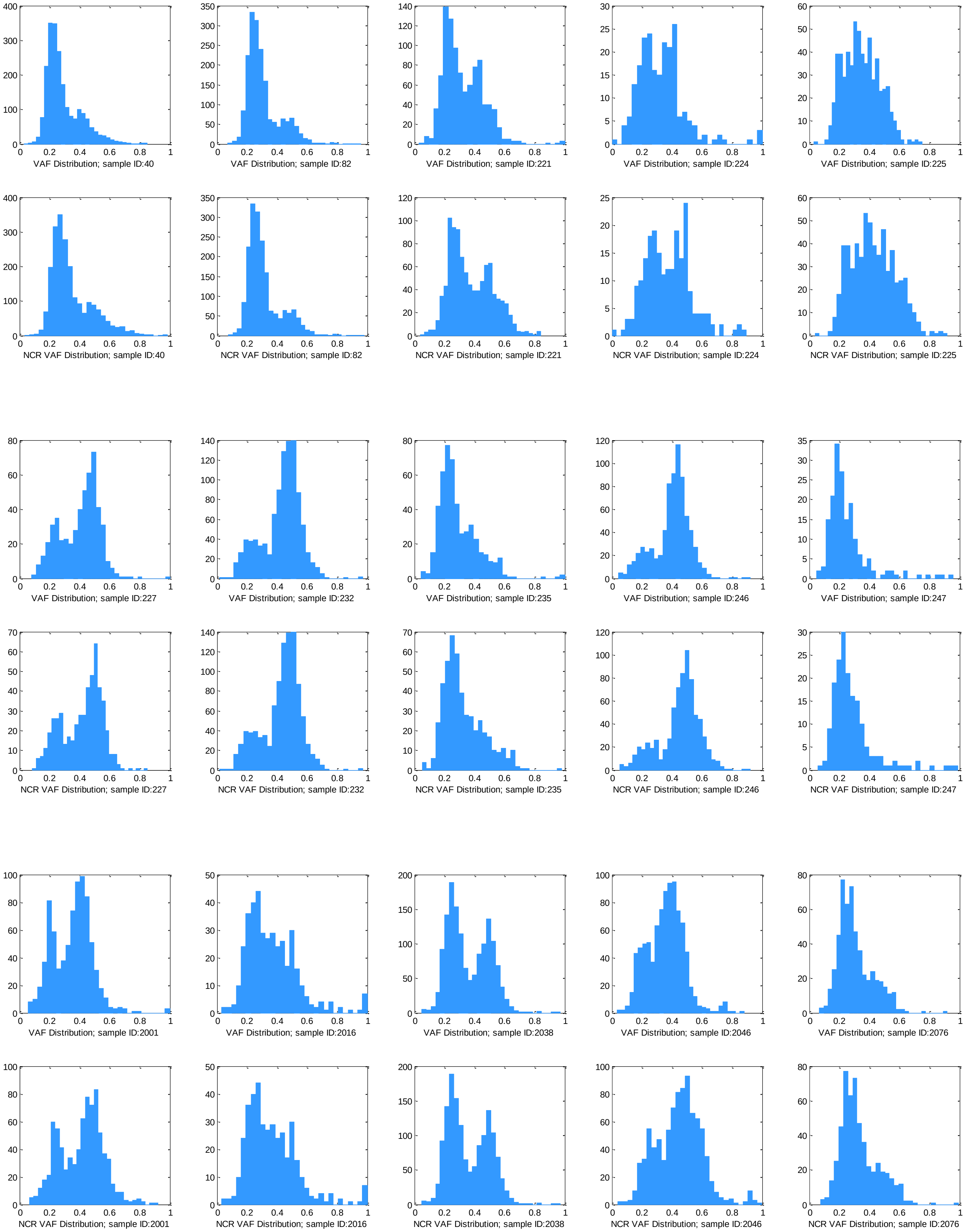

Somatic VAF distribution for Fusion Positive RMS: before and after NCR (normal contamination correction)
Each row has two subrows: the upper one is before NCR the lower one is after NCR

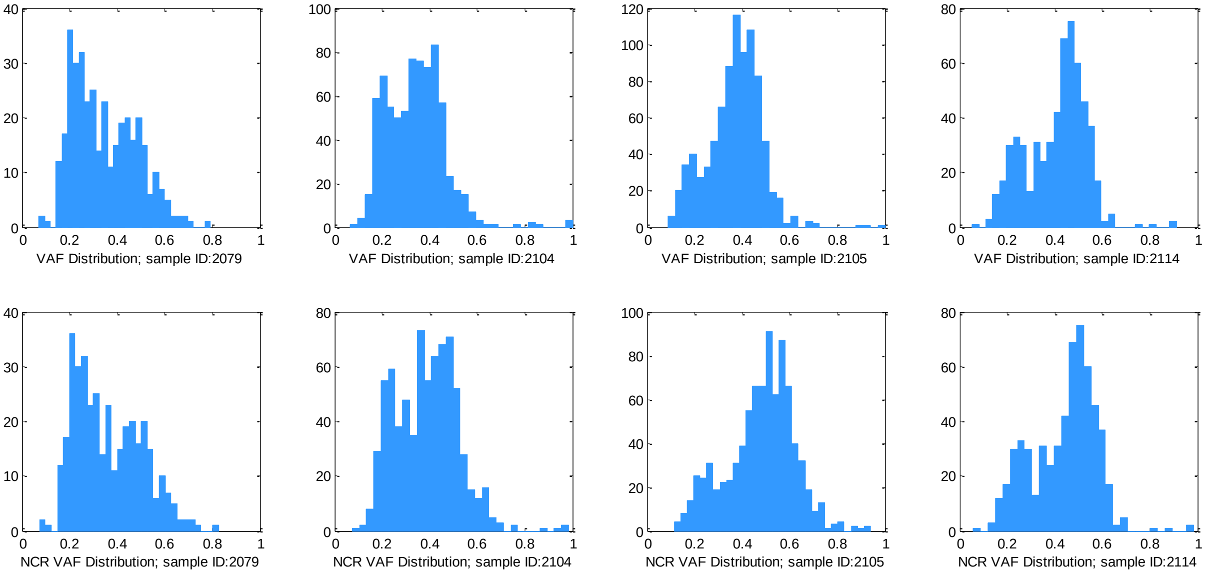

Supplement: S4 Fig — For samples without whole genome duplication (mostly PFN rhabdomyosarcoma), we plot VAF distribution on chromosomes with diploidy “AB”; for samples with whole genome duplication (mostly PFP-RMS), we plot VAF distribution on chromosomes with tetrasomy “AABB”. Each row shows 5 samples. Each row has two sub rows: the upper one shows the VAF distribution before normal cell contamination correction and the lower one shows the distribution after normal cell contamination. We can see that most samples have VAF centered on 0.5 in diploidy chromosomes, or VAF centered on 0.5 and 0.25 on tetrasomy chromosomes. Additionally, for some samples, we observed a small amount of mutations have VAF lower than expected. This finding indicates that these mutations are present only on a part of tumor cells (subclonal mutations). (PDF) [file pgen.1005075.s005.pdf]
