# Supplementary material for: Clonality and Evolutionary History of Rhabdomyosarcoma
Source: PLoS Genet. 2015 Mar 13;11(3):e1005075. doi: 10.1371/journal.pgen.1005075 (PMC4358975; doi:10.1371/journal.pgen.1005075)
Supplement: S7 Fig — (a) Allele “A” gets duplicated at a certain time (indicated by the purple box). If a mutation occurred on allele “A” before the duplication (the pink circle), its mutant copy gets duplicated as well and thus the VAF is 2/3. On the contrary, if a mutation occurred after the duplication (the light green circle), there is only one mutant copy and the VAF is 1/3. The occurrence time of the duplication can be inferred by comparing the number of mutations with VAF = 1/3 to the number of mutations with VAF = 2/3. (b) illustrates another example where the chromosome has an “LOH+duplication” event. (PDF) [file pgen.1005075.s008.pdf]

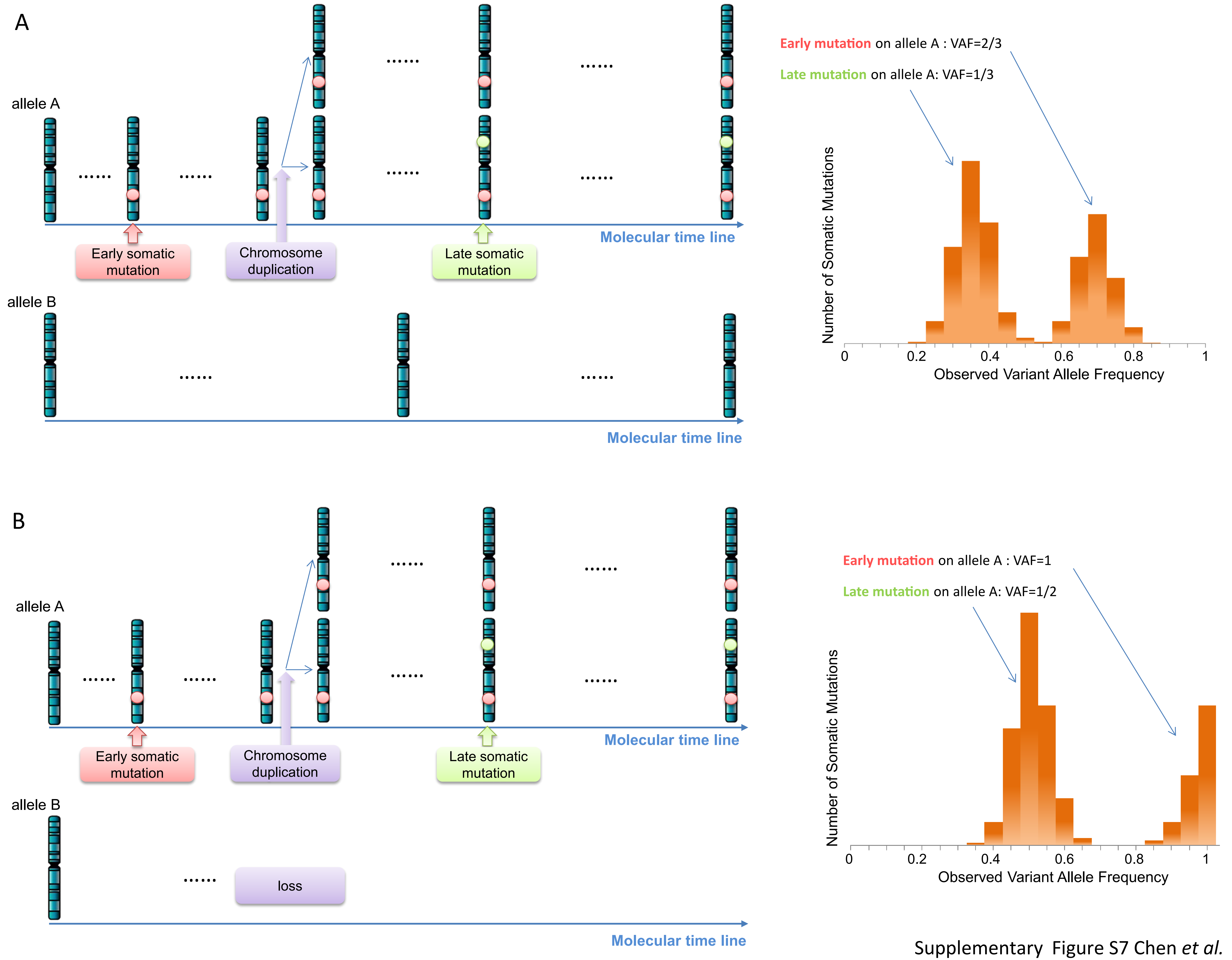

Supplementary Figure S7 Chen *et al.*
