# Supplementary material for: Clonality and Evolutionary History of Rhabdomyosarcoma
Source: PLoS Genet. 2015 Mar 13;11(3):e1005075. doi: 10.1371/journal.pgen.1005075 (PMC4358975; doi:10.1371/journal.pgen.1005075)
Supplement: S14 Fig — (PDF) [file pgen.1005075.s015.pdf]

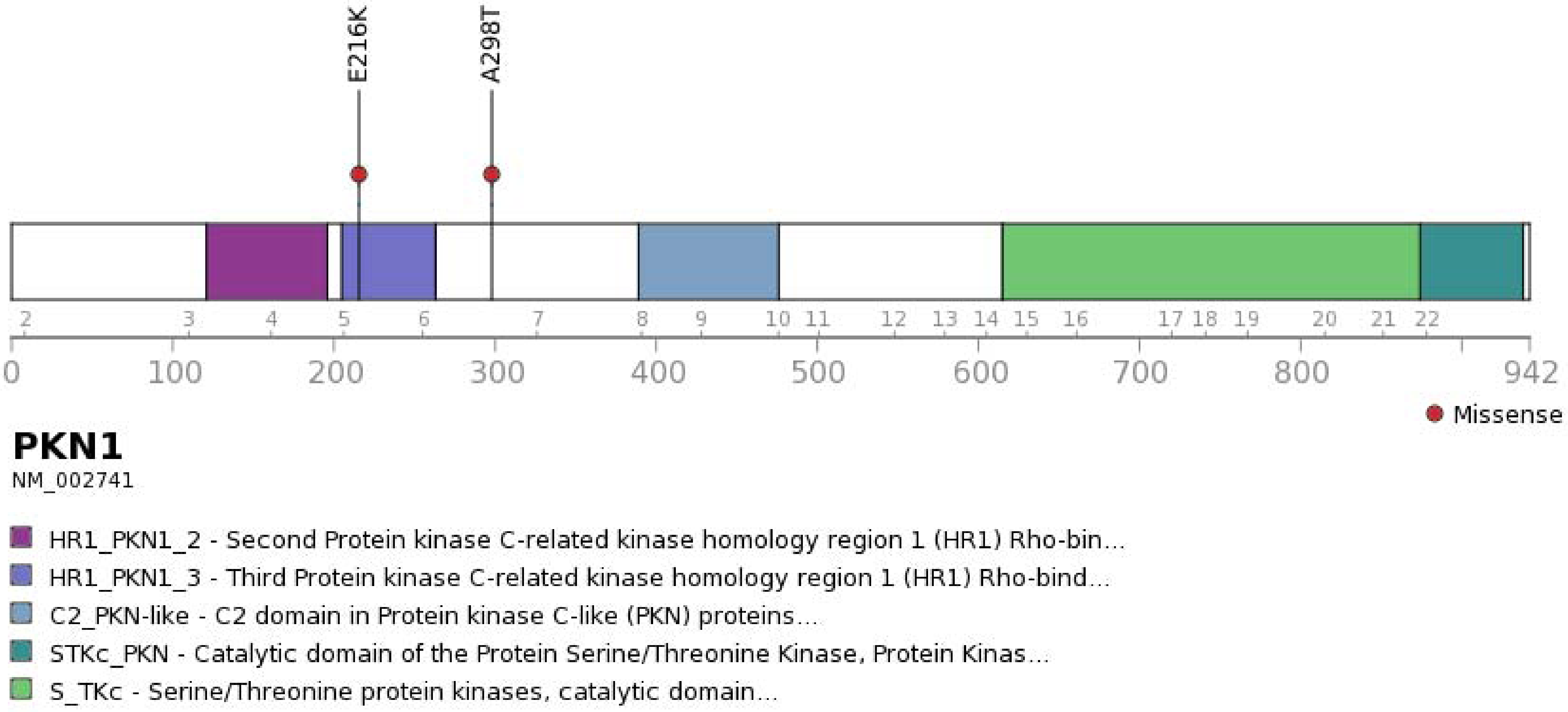

Supplementary Figure S14. Chen *et al.*
